# Anticancer Effects of Broccoli Sprout Extract and Sulforaphane Through ROS-Induced MAPK Pathway in Breast Cancer Cells and Xenografts

**DOI:** 10.3390/antiox15050569

**Published:** 2026-04-30

**Authors:** Seung-On Lee, Ji Eun Yu, Laxman Subedi, Susmita Phuyal, Arjun Dhwoj Bamjan, Goo Yoon, Sang Hoon Joo, Suk-Jung Oh, Jin Woo Park, Jung-Hyun Shim

**Affiliations:** 1 Department of Biomedicine, Health & Life Convergence Sciences, BK21 Four, College of Pharmacy, Mokpo National University, Muan 58554, Republic of Korealaxmansubedi789@gmail.com (L.S.);; 2 Department of Pharmacy, College of Pharmacy, Mokpo National University, Muan 58554, Republic of Korea; 3 College of Pharmacy, Daegu Catholic University, Gyeongsan 38430, Republic of Korea; 4 Research & Development, Ecoworld Pharm Co., Ltd., Damyang 57304, Republic of Korea; 5 The China-US (Henan) Hormel Cancer Institute, Zhengzhou 450008, China

**Keywords:** broccoli sprout extract, sulforaphane, reactive oxygen species, MAPK signaling pathway, oral bioavailability

## Abstract

Breast cancer treatment remains challenging due to therapeutic resistance and the limited availability of effective molecular targets. We investigated the anticancer effects of sulforaphane (SFN) and broccoli sprout extract (BSE), an SFN-enriched phytochemical formulation, in MCF7 and MDA-MB-231 breast cancer cells. Cell viability, colony formation, and apoptotic responses were evaluated using standard *in vitro* assays, and underlying mechanisms were examined by flow cytometry and Western blot analysis. BSE and SFN reduced cell viability in a dose-dependent manner, suppressed anchorage-independent growth, and induced apoptosis associated with increased reactive oxygen species (ROS) generation and activation of c-Jun N-terminal kinase and p38 MAPK signaling pathways. These effects were accompanied by mitochondrial depolarization, G2/M cell cycle arrest, and caspase activation. Pharmacokinetic analysis in rats demonstrated that oral administration of BSE resulted in sustained, dose-dependent systemic exposure to SFN. Consistent with these findings, oral BSE significantly inhibited tumor growth in breast cancer xenograft models. Collectively, these results indicate that BSE exerts anticancer effects through coordinated modulation of ROS-associated MAPK signaling, mitochondrial dysfunction, and apoptotic pathways, and may serve as a promising orally administered SFN-containing phytochemical formulation that may function as a delivery matrix for breast cancer management.

## 1. Introduction

Breast cancer accounts for approximately one-third of all newly diagnosed cancers in women and is the second leading cause of cancer-related mortality among women [1]. Recent epidemiological studies indicate divergent age-specific incidence trends, with increasing rates observed among women younger than 50 years compared to older populations. Breast cancer is a heterogeneous disease comprising multiple molecular subtypes, including luminal A, luminal B, human epidermal growth factor receptor 2 (HER2)-enriched, and triple-negative breast cancer (TNBC), each defined by distinct gene expression profiles and clinical characteristics [2].

Current breast cancer treatments, including surgery, radiotherapy, chemotherapy, endocrine therapy, and targeted approaches, have markedly improved patient survival but remain limited by significant toxicity and the development of therapeutic resistance [2]. Radiotherapy effectively reduces local recurrence yet can injure surrounding healthy tissues, causing long-term complications such as fibrosis and lymphedema [2]. Chemotherapy is indispensable, especially for aggressive subtypes like TNBC, but its nonspecific action leads to severe systemic side effects, including myelosuppression, fatigue, alopecia, infection risk, and chronic organ toxicities such as cardiotoxicity and neurotoxicity. A critical challenge across chemotherapeutic regimens is the frequent emergence of drug resistance, driven by survival signaling activation, increased drug efflux, and tumor genetic alterations, which ultimately results in treatment failure, disease recurrence, and poor prognosis [2]. Likewise, endocrine therapies are initially effective in hormone receptor–positive breast cancer but often lose efficacy over time due to acquired resistance [2]. Collectively, these limitations underscore the urgent need for alternative or adjunct therapeutic strategies that can overcome resistance while reducing toxicity and improving long-term clinical outcomes.

The advent of molecularly targeted therapies has significantly transformed cancer treatment [3]. On the other hand, phytoconstituents comprise a diverse class of bioactive secondary metabolites that exhibit a broad spectrum of pharmacological activities, including antioxidative, anti-inflammatory, and antineoplastic effects [2]. Unlike conventional synthetic chemotherapeutics, which typically exert their effects through single-target mechanisms, phytochemicals demonstrate pleiotropic biological activity, enabling the simultaneous regulation of multiple molecular targets and signaling pathways implicated in carcinogenesis, tumor progression, and therapeutic resistance [2].

Among various factors in cancer, reactive oxygen species (ROS) are highly reactive metabolic intermediates that exert context-dependent oncogenic or tumor-suppressive effects according to their intracellular concentrations, were mostly targeted by phytochemicals such as alkaloids, tannins, flavonoids or other constituents [4,5]. ROS activate c-Jun N-terminal kinase (JNK)/p38 mitogen-activated protein kinase (MAPK) pathways to promote mitochondrial apoptosis *via* caspase activation and induce lipid peroxidation/ER stress leading to ferroptosis or CHOP-mediated apoptosis [4,5,6]. On the other hand, recent studies indicate that phytochemicals influence cancer cells’ behavior by modulating multiple signaling pathways, affecting critical processes such as proliferation, apoptosis, and metastasis [7].

Cruciferous vegetables, such as broccoli, exert chemo-preventive effects through their isothiocyanate derivatives including sulforaphane (SFN) *via* diverse and distinct mechanisms of action such as activation of apoptotic programs, induction of cell-cycle checkpoint arrest, suppression of tumor angiogenesis, and metastatic dissemination [8]. In HepG2 cells, SFN-induced apoptosis has been shown to occur primarily through suppression of the Akt/MAPK signaling pathways and disruption of mitochondrial function [9]. Moreover, SFN inhibits breast cancer progression by modulating key oncogenic pathways, notably Wnt/β-catenin signaling [9]. Consistently, SFN has also been reported to downregulate β-catenin expression in human cervical carcinoma HeLa cells and hepatocellular carcinoma HepG2 cells [10]. In C6 rat glioma model, a dose of 100 mg/kg broccoli sprout extract (BSE) and 0.1 mg/kg SFN administered for 30 days before tumor induction effectively prevented tumor development [11]. Furthermore, preclinical studies have demonstrated that SFN treatment effectively inhibits the proliferation and self-renewal of breast cancer cells in animal models, suggesting its potential to modulate key oncogenic pathways *in vivo* [9]. Despite accumulating evidence suggesting that BSE possesses broad anticancer potential, BSE alone has not been tested for its anticancer activity yet [12,13,14,15]. Accordingly, the mechanisms by which BSE suppresses breast cancer metastasis and the molecular pathways involved have yet to be clearly elucidated.

Thus, we determined the anticancer efficacy of BSE in breast cancer cells, and characterized the underlying mechanisms. In addition, we characterized the oral pharmacokinetics of BSE using SFN as a reference bioactive constituent and evaluated its antitumor efficacy in breast cancer xenograft models, as well as MCF7 and MDA-MB-231 cells. While pure SFN is a potent bioactive compound, its clinical use is limited by rapid systemic clearance and poor pharmacokinetic retention. In contrast, BSE may offer a practical advantage as a natural delivery matrix, warranting pharmaco-kinetic evaluation of both BSE and pure SFN.

## 2. Materials and Methods

### 2.1. Materials

SFN (purity > 96%) was purchased from ApexBio Technology (Houston, TX, USA). Dulbecco’s modified Eagle medium (DMEM) was obtained from Welgene (Gyeongsan, Republic of Korea). The 3-(4,5-dimethylthiazol-2-yl)-2,5-diphenyltetrazolium bromide (MTT) reagent was purchased from Biosesang (Yongin, Republic of Korea). The hematoxylin and eosin (H&E) staining kit was obtained from Abcam (Cambridge, UK). Enhanced chemiluminescence reagent was purchased from Thermo Fisher Scientific (Waltham, MA, USA). Polyvinylidene difluoride (PVDF) membranes were obtained from Millipore (Billerica, MA, USA).

### 2.2. Preparation of BSE

Based on previously reported methods for SFN-rich BSE [16], BSE was prepared using a water-based endogenous extraction process. Fresh broccoli sprouts were washed, dried at 40–45 °C for 48 h, milled into a powder, and stored at 20 °C under controlled relative humidity (60 ± 5%) prior to extraction. The powdered sprouts were suspended in distilled water (10 g/100 mL) and incubated at 37 °C for 60 min. This incubation step was specifically included to promote the conversion of glucoraphanin to SFN through the hydrolytic action of residual endogenous myrosinase released upon tissue disruption, rather than through any chemical modification process. The suspension was then extracted at 25 °C for 3 h. Although myrosinase activity was not directly quantified after the pretreatment steps, the measurable formation of SFN during incubation suggests that sufficient endogenous enzymatic activity was retained under the relatively mild drying and storage conditions used. Therefore, the conversion of glucoraphanin to SFN in this system is interpreted to be primarily enzyme-mediated. After filtration, the aqueous extract was concentrated and lyophilized, and the resulting BSE powder was stored at −20 °C until use.

The extraction process was performed in independent batches under identical conditions, and comparable extraction yields and SFN contents were obtained, indicating consistent extraction yields and SFN contents across independent batches. The extraction yield was approximately 9.8 ± 1.2% (*w*/*w*) relative to the starting dried material. Importantly, although SFN is enriched during this process, the final product is not a purified compound but a chemically complex phytochemical extract containing multiple coexisting constituents. The SFN content in BSE was quantified using a previously established chromatographic method [16]. Briefly, BSE samples were dissolved in methanol–water (1:1, *v*/*v*), filtered through a 0.45 μm membrane filter, and analyzed by high-performance liquid chromatography (HPLC) with ultraviolet detection at 202 nm using comparison with an authentic SFN standard. The analytical method demonstrated strong linearity over the tested concentration range (r^2^ = 0.9997) with a limit of detection (LOD) of 25 ppm, as reported in our previous study [16]. In the present work, this established method was used for quantification and standardization of SFN content in the lyophilized BSE. The SFN content values used for *in vivo* dose calculation were based on the quantified SFN content of the lyophilized extract determined using this method, allowing conversion of extract dosing into SFN-equivalent exposure.

### 2.3. Cell Culture

Human breast cancer cell lines MDA-MB-231 (HTB-26) and MCF7 (HTB-22), and the human keratinocyte cell line HEKa (PCS 200-011) were obtained from the American Type Culture Collection (Manassas, VA, USA). Cells were cultured in DMEM supplemented with 10% fetal bovine serum (Corning, NY, USA) and 100 U/mL penicillin–streptomycin (Thermo Fisher Scientific) in a humidified incubator (95% air and 5% CO_2_) at 37 °C.

### 2.4. MTT Assay

MCF7 (1 × 10^5^/mL per well), MDA-MB-231 (4 × 10^4^/mL per well), and HEKa (8 × 10^4^/mL per well) cells were seeded in 96-well plates and treated with BSE or SFN at the indicated concentrations (BSE: 80 or 160 μg/mL; SFN: 1 μg/mL) for 24 h or 48 h. For inhibitor studies (SP600125, SB203580, NAC, Z-VAD-FMK), cells were pretreated with the indicated inhibitors for 3 h prior to treatment with BSE or SFN. Cell viability was assessed using the MTT assay as described previously [17]. Absorbance was measured at 570 nm using a microplate reader (Thermo Fisher Scientific).

### 2.5. Western Blot Analysis

Following treatment with SFN or BSE, cells and excised tumor tissues were homogenized using radioimmunoprecipitation assay (RIPA) buffer (Intron Biotechnology, Seongnam, Republic of Korea) containing 0.1 mM phenylmethylsulfonyl fluoride (PMSF), 10 μg/mL leupeptin, and 10 μg/mL aprotinin. Protein extracts were separated by sodium dodecyl sulfate–polyacrylamide gel electrophoresis and transferred to PVDF membranes (Merck-Millipore, Burlington, MA, USA). Western blotting was performed as described previously [17]. Primary antibodies were purchased from Santa Cruz Biotechnology (Dallas, TX, USA) or Cell Signaling Technology (Danvers, MA, USA). Antibodies against phospho–SAPK/c-Jun N-terminal kinase (p-JNK; 46, 54 kDa), phospho-p38 (p-p38; 43 kDa), JNK (46, 54 kDa), and p38 (43 kDa) were obtained from Cell Signaling Technology. Antibodies against p21 (21 kDa), p27 (27 kDa), cyclin B1 (55 kDa), cell division cycle 2 (cdc2; 34 kDa), glucose-regulated protein 78 (GRP78; 78 kDa), BCL2-associated agonist of cell death (Bad; 25 kDa), myeloid cell leukemia 1 (Mcl-1; 32, 40 kDa), BH3-interacting domain death agonist (BID; 22 kDa), BCL2-associated X protein (Bax; 23 kDa), B-cell lymphoma-extra large (Bcl-xL; 30 kDa), B-cell lymphoma 2 (Bcl-2; 26 kDa), cytochrome c (cyt c; 15 kDa), β-tubulin (55 kDa), cytochrome c oxidase subunit 4 (COX4; 17 kDa), apoptosis-activating factor 1 (Apaf-1; 130 kDa), caspase3 (32 kDa), poly(ADP-ribose) polymerase (PARP; 89, 116 kDa), DNA damage-inducible transcript 3/growth arrest and DNA damage-inducible protein 153 (DDIT3/GADD153/CHOP; CHOP; 30 kDa), and β-actin (43 kDa) were obtained from Santa Cruz Biotechnology. The intensities of the immunoreactive bands were quantified by densitometry using ImageJ software (version 1.4.3.67, National Institutes of Health, Bethesda, MD, USA). The levels of phosphorylated proteins were normalized to the corresponding total protein levels, and β-actin was used as an internal control for overall protein loading normalization.

### 2.6. Mitochondria and Cytosolic Fractionation

Enriched cytosolic and mitochondrial fractions were isolated from BSE- or SFN-treated breast cancer cells using plasma membrane extraction (PME) buffer (250 mM sucrose, 10 mM HEPES, 10 mM potassium chloride, 1.5 mM magnesium chloride hexahydrate, 1 mM ethylenediaminetetraacetic acid [EDTA], 1 mM ethylene glycol-bis(β-aminoethyl ether)-N,N,N′,N′-tetraacetic acid [EGTA], 0.01 mg/mL leupeptin, 0.01 mg/mL aprotinin, and 0.1 mM PMSF). Cells were harvested and homogenized using ice-cold RIPA buffer supplemented with 0.1% digitonin, vortexed for 1 min, and incubated at room temperature (RT) for 10 min. The homogenate was centrifuged at 13,000 rpm, 4 °C, for 5 min, and the supernatant was collected as the cytosolic fraction. The pellet was washed twice, resuspended in PME buffer, and centrifuged under the same conditions. The resulting supernatant was treated with 0.5% Triton X-100, incubated on ice for 10 min with gentle tapping every 3 min, and centrifuged at 13,000 rpm, 4 °C, for 30 min. The supernatant was collected as the mitochondrial fraction. Expression of cyt c in cytosolic and mitochondrial fractions was assessed by Western blotting. COX4 and β-tubulin were used as markers for the mitochondrial and cytosolic fractions, respectively.

### 2.7. Anchorage-Independent Soft Agar Assay

Anchorage-independent growth was evaluated using a soft agar colony formation assay. A bottom layer was prepared by mixing 0.6% low-melting agarose (Thermo Fisher Scientific) with Basal Medium Eagle (BME; Sigma-Aldrich, St. Louis, MO, USA) and added to six-well plates to solidify at RT. For the upper layer, cells were resuspended at 8000 cells/mL in 0.6% agarose dissolved in complete growth medium containing the indicated treatments (1 μg/mL SFN or 160 μg/mL BSE) and overlaid on the solidified bottom layer. Plates were incubated at 37 °C in a humidified 5% CO_2_ atmosphere for 15 days (MCF7) or 28 days (MDA-MB-231). Colonies were analyzed under an inverted microscope (Leica, Wetzlar, Germany), and only colonies with diameters > 50 μm were counted. All experiments were performed in triplicate to ensure reproducibility.

### 2.8. Cell Apoptosis Analysis by Flow Cytometry

MCF7 and MDA-MB-231 cells were seeded in six-well plates at densities of 1 × 10^4^ cells/mL and 4 × 10^3^ cells/mL, respectively, and allowed to adhere overnight. Next, the cells were treated with SFN (1 μg/mL) or BSE (80 μg/mL or 160 μg/mL) for 48 h. Following treatment, both adherent and floating cells were collected, washed twice with phosphate-buffered saline (PBS), and resuspended in 1× binding buffer. Subsequently, the cells were stained with 5 μL of annexin V-FITC and 5 μL of 7-amino-actinomycin D (7-AAD) using a Muse Annexin V & Dead Cell Kit (Cytek Biosciences, Fremont, CA, USA). The stained cells were incubated in the dark for 15 min at RT. Apoptosis was assessed using flow cytometry (Muse Cell Analyzer; Merck Millipore, Burlington, MA, USA). Data were processed using Muse analysis software (SN7200120642) to determine the proportions of live, early apoptotic, late apoptotic, and necrotic cells. All experimental conditions were performed in triplicate to ensure reproducibility and statistical reliability.

### 2.9. Cell Cycle Analysis

Cells were treated with SFN and BSE under the same conditions described for the apoptosis assay. After treatment, cells were collected by trypsinization, washed twice with cold PBS, and fixed in 70% ethanol at −20 °C for at least 24 h. Fixed cells were centrifuged at 300× *g* for 5 min, the ethanol was removed, and the cell pellets were resuspended in a staining solution containing 50 μg/mL propidium iodide (PI) and 100 μg/mL RNase A in PBS. The cells were stained in the dark at RT for 30 min. Cell cycle distribution was assessed using a MACSQuant Analyzer 16 (Miltenyi Biotec, Bergisch Gladbach, Germany), recording at least 30,000 events per sample.

### 2.10. Intracellular ROS Detection

Intracellular ROS levels were measured using 2′,7′-dichlorodihydrofluorescein diacetate (H2DCFDA; Sigma-Aldrich), a cell-permeable fluorogenic probe. MCF7 and MDA-MB-231 cells were seeded in six-well plates at densities of 1 × 10^4^ cells/mL and 4 × 10^3^ cells/mL, respectively, and allowed to adhere overnight. The cells were treated with SFN (1 μg/mL) or BSE (80 μg/mL and 160 μg/mL) for 48 h. After treatment, the cells were washed twice with PBS and incubated with 10 μM H2DCFDA in serum-free medium at 37 °C for 30 min in the dark. Next, the cells were harvested using trypsin/EDTA, centrifuged at 300× *g* for 5 min, and resuspended in PBS. The fluorescence intensity of the oxidized product, dichlorofluorescein, was immediately measured using a MACSQuant Analyzer with excitation at 488 nm and emission at 530 nm.

### 2.11. Mitochondrial Membrane Potential (MMP) Measurement

MMP was assessed using JC-1 dye (Thermo Fisher Scientific). Harvested cells were washed twice with ice-cold PBS and incubated with JC-1 working solution (10 μg/mL in PBS) at 37 °C for 20 min in the dark. After staining, the cells were washed twice with JC-1 staining buffer and resuspended in PBS. Fluorescence was measured using a MACSQuant Analyzer flow cytometer, recording green fluorescence for JC-1 monomers and red fluorescence for JC-1 aggregates. The ratio of red to green fluorescence intensity was used to quantify MMP.

### 2.12. Caspase Activity Assay

Multi-caspase activity was measured using a Muse Multi-Caspase Assay Kit (Cytek Biosciences). MCF7 and MDA-MB-231 cells were seeded in six-well plates at densities of 1 × 10^4^ cells/mL and 4 × 10^3^ cells/mL, respectively, and allowed to adhere overnight. The cells were treated with SFN (1 μg/mL) or BSE (80 μg/mL and 160 μg/mL) for 48 h. Following treatment, the cells were processed as described previously [17]. Fluorescence intensity was measured using a Muse Cell Analyzer (Cytek Biosciences), and caspase activity was expressed as the percentage of positive cells.

### 2.13. Animals

Male Sprague–Dawley rats (6–7 weeks old; 200–250 g) and female BALB/c nude mice (BALB/c nu/nu, 6 weeks old; 18–22 g at randomization) were obtained from a certified commercial supplier (G-bio, Gwangju, Republic of Korea). Male Sprague–Dawley rats were used exclusively for the pharmacokinetic study, whereas female BALB/c nude mice were used exclusively for the xenograft antitumor efficacy study. The rat model was selected for pharmacokinetic analysis because repeated serial blood sampling required for full plasma concentration–time profiling is technically more feasible and reliable in rats than in mice. Accordingly, the pharmacokinetic study was designed to evaluate systemic exposure and oral absorption of SFN, whereas the mouse xenograft model was used independently to assess *in vivo* antitumor efficacy. Animals were housed under controlled conditions, with a temperature of 23 ± 2 °C, relative humidity of 55 ± 10%, and a 12 h light/dark cycle. All animals had ad libitum access to standard laboratory chow (Nestlé Purina PetCare Research, St. Louis, MO, USA) and ion-sterilized drinking water. All animal experiments were performed in accordance with the relevant institutional and national guidelines for the care and use of laboratory animals. All experimental procedures were reviewed and approved by the Institutional Animal Care and Use Committee (IACUC) of Mokpo National University (Jeonnam, Republic of Korea; approval numbers MNU-IACUC-2025-012 and MNU-IACUC-2025-013).

### 2.14. In Vivo Oral Absorption of SFN in Rats

The pharmacokinetics of SFN administered as a purified compound or within an SFN-rich BSE were evaluated exclusively in male Sprague–Dawley rats. Rats were selected for pharmacokinetic analysis because serial blood sampling required for full plasma concentration–time profiling is technically more feasible and reliable in rats than in mice, in which repeated sampling can impose substantial physiological burden and increase experimental variability. Accordingly, this study was designed to assess systemic exposure and oral absorption behavior of SFN after BSE administration, rather than to establish a species- and sex-matched pharmacokinetics/pharmacodynamics (PK/PD) model for the mouse xenograft study. Male rats were used to reduce variability associated with hormonal fluctuations and to ensure consistent pharmacokinetic evaluation.

The selected dose levels were determined based on previously reported SFN pharmacokinetic studies and our previous *in vivo* study using the same BSE platform, in which an SFN-equivalent dose of approximately 5 mg/kg showed biological activity [18]. Accordingly, this reference dose and additional higher doses were included to evaluate dose-dependent pharmacokinetic behavior. Animals received SFN *via* oral or intravenous (IV) routes according to the study design. For oral administration, free SFN was given at 5 mg/kg as a suspension in 0.5% (*w*/*v*) sodium carboxymethyl cellulose, whereas BSE formulations prepared in water were administered at SFN-equivalent doses of 5, 10, or 20 mg/kg (800 µL per dose). To estimate absolute oral bioavailability, a reference IV group received SFN at 1 mg/kg in normal saline *via* femoral vein cannulation (200 µL injection volume). Serial blood samples were collected at predetermined intervals following dosing. For IV-treated animals, samples were obtained at 0.17, 0.5, 1, 1.5, 2, 4, 8, 12, and 24 h post-administration; for orally dosed animals, samples were collected at 0.17, 0.5, 1, 2, 4, 6, 8, 12, and 24 h. Approximately 200 µL of blood was collected at each time point and immediately centrifuged at 13,000× *g* for 5 min. Plasma was separated and stored at −80 °C until bioanalysis. Each pharmacokinetic group consisted of three animals (*n* = 3).

Plasma SFN concentrations were quantified using a validated ultra-performance liquid chromatography–tandem mass spectrometry (UPLC–MS/MS) method following protein precipitation and solid-phase extraction. Plasma aliquots (180 µL) were spiked with calibration standards (0.2–20 µg/mL SFN) and the internal standard phenethyl isothiocyanate (5 µg/mL in acetonitrile). Proteins were precipitated by adding 200 µL of a mixed organic solvent (ethanol/acetonitrile/water, 50:25:25, *v*/*v*/*v*), followed by centrifugation at 14,000 rpm for 5 min. Subsequently, the supernatant (400 µL) was subjected to cleanup using hydrophilic–lipophilic balance µElution solid-phase extraction plates preconditioned with methanol and water. After sequential washing with water and 50% aqueous methanol, analytes were eluted with 400 µL of acetonitrile (200 µL × 2). Chromatographic separation was performed on an ACQUITY UPLC system equipped with a BEH C18 analytical column (100 × 2.1 mm, 1.7 µm). The mobile phase consisted of 10 mM ammonium acetate in water (30%, *v*/*v*) and acetonitrile containing 0.1% formic acid (70%, *v*/*v*), delivered at a flow rate of 0.6 mL/min. The column temperature was maintained at 50 °C, and the injection volume was 10 µL. Detection was performed using a Xevo TQ-S triple quadrupole mass spectrometer (Waters Corporation, Milford, MA, USA) operating in positive electrospray ionization mode with multiple reaction monitoring (MRM). The monitored precursor-to-production transitions were *m*/*z* 178.0 → 114.0 for SFN and *m*/*z* 164.0 → 130.0 for the internal standard. Optimized source parameters included a capillary voltage of 3.0 kV, source temperature of 120 °C, desolvation temperature of 500 °C, desolvation gas flow of 800 L/h, and cone gas flow of 50 L/h. All MRM transitions and instrument settings were optimized using MassLynx software (version 4.2; Waters Corporation).

### 2.15. In Vivo Anticancer Efficacy of BSE in MCF7 and MDA-MB-231 Xenograft Models

The antitumor efficacy of orally administered BSE was evaluated independently of the rat pharmacokinetic study using breast cancer xenograft models established in female BALB/c nude mice. Whereas the separate rat study was designed to confirm systemic exposure and oral absorption of SFN after BSE administration, the mouse xenograft study was performed specifically to evaluate *in vivo* antitumor efficacy. To capture distinct biological and molecular tumor contexts, MCF7 cells, representing hormone-dependent, estrogen/progesterone receptor–positive luminal tumors with relatively low aggressiveness, and MDA-MB-231 cells, a highly invasive TNBC model with basal-like characteristics, were selected for xenograft implantation in female BALB/c nude mice (BALB/c nu/nu, 7 weeks old). To establish a favorable hormonal environment for MCF7 xenograft formation, estradiol valerate was administered subcutaneously at 4 mg/kg once daily for 2 days prior to tumor implantation, followed by continued administration once every 3 days until the end of the experiment. Next, MCF7 cells (7 × 10^6^ cells in 0.1 mL PBS, pH 7.4) were injected subcutaneously into the right dorsal flank. For MDA-MB-231 xenografts, cells (7 × 10^6^ in 100 µL PBS, pH 7.4, containing 0.5 mg/mL Matrigel) were inoculated subcutaneously into 7-week-old mice. Once tumors reached approximately 100 mm^3^, animals were randomly assigned to four treatment groups for each cell type (*n* = 10 per group) using a predefined randomization scheme: Control (vehicle only water, oral, once daily), BSE (25) (0.625 mg/kg SFN; 25 mg/kg BSE, oral, once daily), BSE (50) (1.25 mg/kg SFN; 50 mg/kg BSE, oral, once daily), and BSE (100) (2.5 mg/kg SFN; 100 mg/kg BSE, oral, once daily). The selected dose levels were determined based on the SFN content of the extract and previously reported SFN efficacy-related dose ranges. Previous studies have demonstrated that SFN exhibits *in vivo* biological and anticancer activity at oral doses in the range of approximately 1–10 mg/kg, depending on the experimental model [19,20]. In our previous study using the same BSE platform, an SFN-equivalent dose of approximately 5 mg/kg showed *in vivo* biological activity [18]. Based on these findings, the present study employed a lower SFN-equivalent dose range (0.625–2.5 mg/kg) to evaluate dose-dependent antitumor efficacy under repeated oral administration conditions. This dosing strategy was further supported by pharmacokinetic results demonstrating sustained systemic exposure of SFN following oral BSE administration. A conventional cytotoxic positive control group was not included in this study because the primary objective was to evaluate the intrinsic antitumor efficacy of orally administered BSE rather than to directly compare it with standard chemotherapeutic agents. Bodyweight and tumor volume were recorded every 3 days. Tumor measurements and data analysis were performed in a blinded manner to minimize experimental bias, and investigators involved in tumor measurement were blinded to treatment allocation. Tumor volume was calculated using the standard ellipsoid formula: (width)2 × length × 0.52. To minimize measurement variability, tumor volumes were assessed using consistent caliper-based methods by the same investigator throughout the study. After 36 or 21 days of treatment, the mice were euthanized, and tumors were excised and weighed for comparative analysis. The treatment duration was selected on the basis of tumor growth kinetics in each xenograft model and was considered sufficient to evaluate dose-dependent antitumor effects while avoiding excessive tumor burden.

### 2.16. Histological Analysis and Immunohistochemistry

H&E staining was performed using an H&E Staining Kit (ab245880, Abcam). Mouse tumor tissues were excised and immediately fixed in 4% formaldehyde, dehydrated through a graded ethanol series (70–100%), and embedded in paraffin. Tumor sections (5 μm) were cut, stained with H&E, and examined under a light microscope (Olympus, Tokyo, Japan).

For immunohistochemistry, paraffin-embedded tumor sections were dewaxed and subjected to antigen retrieval using 1× citrate buffer (pH 6.0; Antigen Retriever, C9999, Sigma-Aldrich) in a microwave oven. After cooling, endogenous peroxidase activity was blocked with hydrogen peroxide. Sections were blocked with 3% normal goat serum in PBS and incubated with primary antibodies against p-JNK, p-p38, and C/EBP homologous protein (CHOP) at appropriate dilutions in blocking solution. Immunoreactivity was detected using an avidin–biotin–peroxidase complex (ABC; #PK-6101, #PK-6102, Vector Laboratories, Newark, CA, USA) according to the manufacturer’s protocol and visualized with diaminobenzidine substrate (#SK-4100, Vector Laboratories). Stained sections were examined under a light microscope.

### 2.17. Terminal Deoxynucleotidyl Transferase dUTP Nick End Labeling (TUNEL) Assay

Apoptotic cell death in tumor tissue sections was assessed using an *In Situ* Apoptosis Detection Kit (Click-iT™ Plus, C10617, Thermo Fisher Scientific). DNA fragmentation was detected in paraffin-embedded samples using TUNEL. Nuclei were counterstained using 4′,6-diamidino-2-phenylindole (DAPI). TUNEL-positive cells were visualized under a fluorescence microscope. For quantification, TUNEL-positive cells were counted in at least five randomly selected fields per section, and the percentage of TUNEL-positive cells was calculated relative to the total number of DAPI-stained nuclei.

### 2.18. Statistical Analysis

Data are presented as the mean ± standard deviation (SD) from replicate wells of repeated experiments. Differences between groups were evaluated using one-way or two-way analysis of variance followed by Tukey’s post hoc test or Student’s *t*-test, as appropriate. *P*-values < 0.05 were considered statistically significant. All experiments were performed in triplicate unless stated otherwise. IC_50_ values were calculated using GraphPad Prism software (version 11.0.0; GraphPad Software, San Diego, CA, USA) based on three independent experiments. The results are presented as mean ± standard deviation (SD).

Specific statistical tests used for each figure are indicated in the corresponding figure legends.

## 3. Results

### 3.1. SFN Content of BSE

Quantitative analysis confirmed that BSE contained substantial amounts of SFN, indicating efficient endogenous conversion from its precursor glucoraphanin during the extraction process, which is interpreted to occur primarily through endogenous myrosinase activity. SFN was identified by comparison with an authentic standard and quantified using a previously established chromatographic method with confirmed calibration linearity. The SFN content measured during extraction optimization was approximately 420 ± 25 ppm (µg/g, fresh weight basis of the starting broccoli sprout material, *n* = 3), consistent with previously reported values obtained using the same endogenous extraction approach [16]. This value reflects the SFN yield relative to the original plant material and is consistent with effective enzymatic conversion under the selected processing conditions. In contrast, for the *in vivo* studies, the lyophilized BSE used in this work exhibited a substantially higher SFN content of approximately 25 mg/g (2.5% [*w*/*w*], *n* = 3), reflecting the concentration of SFN during the extraction and lyophilization processes. Accordingly, SFN-equivalent dosing in subsequent animal experiments was determined based on this experimentally measured SFN content of the lyophilized extract. These results clearly distinguish between SFN content expressed on a fresh material basis and that of the final lyophilized extract, thereby avoiding ambiguity in interpretation and ensuring accurate dose conversion. The consistency of SFN content across independent preparations and agreement with previously reported values indicate that the extraction process is reproducible under the defined conditions. In addition, the maintained SFN levels during sample handling and analysis suggest that the extract provides sufficient stability for experimental use under controlled conditions. Importantly, BSE is not a single-compound preparation but a chemically complex phytochemical extract. Previous liquid chromatography–tandem mass spectrometry (LC-MS/MS) and feature-based molecular networking analyses of the same BSE platform have demonstrated the presence of diverse compound classes, including amino acids, alkaloids, organic acids, fatty acids, glycosides, terpenes, sesquiterpenoids, and glucosinolate-derived isothiocyanates [18].

Collectively, these results indicate that BSE represents an SFN-enriched but compositionally complex extract in which the bioactive compound is generated and maintained within a phytochemical matrix, providing a basis for subsequent pharmacokinetic and anticancer efficacy studies.

### 3.2. BSE and SFN Inhibit Cell Viability and Colony Formation in Breast Cancer Cells

**Figure 1:**
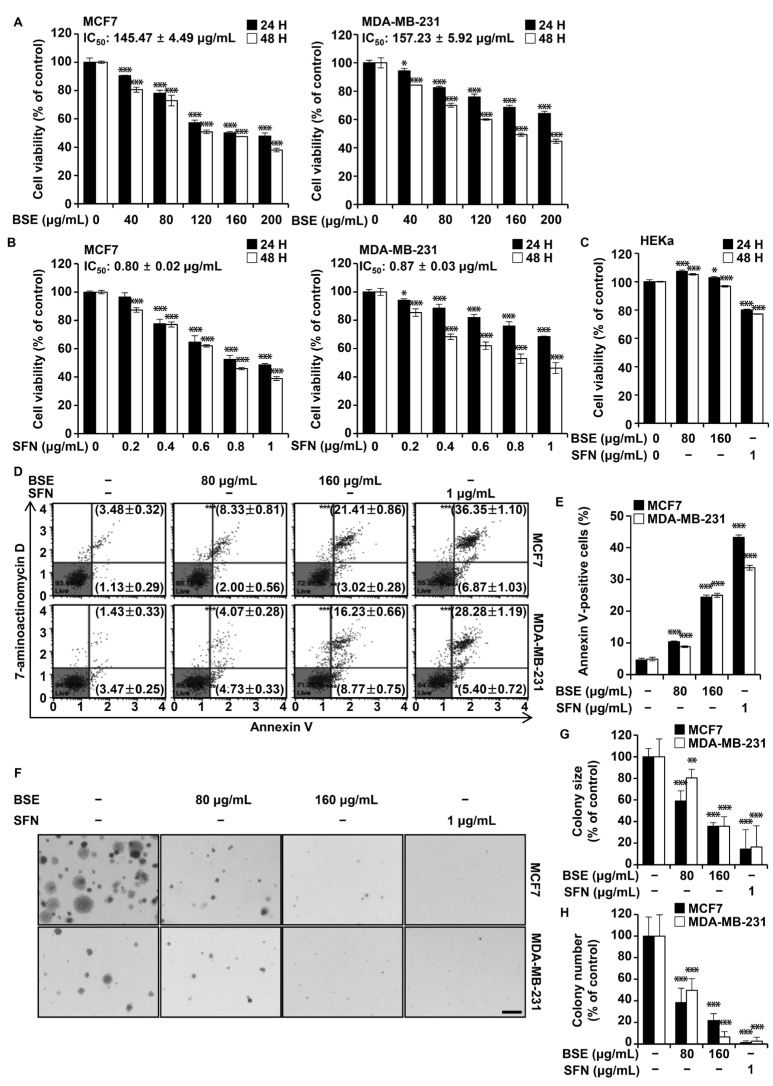
Broccoli sprout extract (BSE) and sulforaphane (SFN) reduced MCF7 and MDA-MB-231 cell viability, induced apoptosis, and inhibited colony formation. (**A**) MCF7 and MDA-MB-231 cells were treated with BSE (0–200 μg/mL) for 24 or 48 h, and cell viability was assessed using the MTT assay. (**B**) MCF7 and MDA-MB-231 cells were treated with SFN (0–1 μg/mL) for 24 or 48 h, and viability was measured. (**C**) Human epidermal keratinocytes were treated with BSE (0, 80, and 160 μg/mL) or SFN (1 μg/mL) for 24 or 48 h, and viability was assessed. (**D**,**E**) MCF7 and MDA-MB-231 cells were treated with BSE or SFN for 48 h, followed by flow cytometry analysis using Muse Annexin V and 7-AAD staining to evaluate apoptosis. (**F**–**H**) Effects of BSE and SFN on colony formation in MCF7 and MDA-MB-231 cells (scale bar, 400 μm). Quantitative data are presented as mean ± standard deviation (*n* = 3). Data were analyzed by one-way ANOVA followed by Tukey’s post hoc test. * *p* < 0.05, ** *p* < 0.01, *** *p* < 0.001 versus vehicle-treated cells.

To evaluate the cytotoxic effects of BSE or SFN, MCF7 and MDA-MB-231 breast cancer cells were treated with BSE (0, 40, 80, 120, 160, and 200 μg/mL) or SFN (0, 0.2, 0.4, 0.6, 0.8, and 1 μg/mL) for 24 or 48 h. Cell viability, assessed using the MTT assay, decreased in a dose-dependent manner following treatment with either BSE or SFN. After 48 h, the half-maximal inhibitory concentration (IC_50_) values for BSE and SFN were 145.47 ± 4.49 µg/mL and 0.80 ± 0.02 µg/mL, respectively, in MCF7 cells, and 157.23 ± 5.92 µg/mL and 0.87 ± 0.03 µg/mL, respectively, in MDA-MB-231 cells (Figure 1A,B). The IC_50_ values were determined using non-linear regression analysis (curve fit) *via* GraphPad Prism software. In contrast, the viability of HEKa cells was not affected by BSE at concentrations up to 160 µg/mL, whereas a slight reduction in viability was observed following SFN treatment at 1.0 µg/mL (Figure 1C). To assess apoptosis, MCF7 and MDA-MB-231 cells were treated with BSE or SFN for 48 h and analyzed by flow cytometry using Annexin V/7-aminoactinomycin D double staining (Figure 1D,E). In MCF7 cells, the proportion of early apoptotic cells (lower right quadrant) increased from 1.13% in controls to 2.00%, 3.02%, and 6.87% following treatment. Corresponding increases in MDA-MB-231 cells were observed from 3.47% to 4.73%, 8.77%, and 5.40%. The proportion of late apoptotic cells (upper right quadrant) in MCF7 cells increased from 3.48% to 8.33%, 21.41%, and 36.35%, whereas in MDA-MB-231 cells, these values increased from 1.43% to 4.07%, 16.23%, and 28.28%. Colony formation assays were performed using BSE at 80 µg/mL and 160 µg/mL or SFN at 1.0 µg/mL. Colony number and size were evaluated on day 15 for MCF7 cells and on day 28 for MDA-MB-231 cells (Figure 1F–H). Both BSE and SFN markedly reduced colony formation in both breast cancer cell lines. These findings demonstrate that in breast cancer cells BSE and SFN inhibit cell viability, induce apoptosis, and suppress long-term clonogenic growth.

### 3.3. BSE and SFN Induce ROS Production and Mitogen-Activated Protein Kinase (MAPK)-Mediated Apoptosis in Breast Cancer Cells

**Figure 2:**
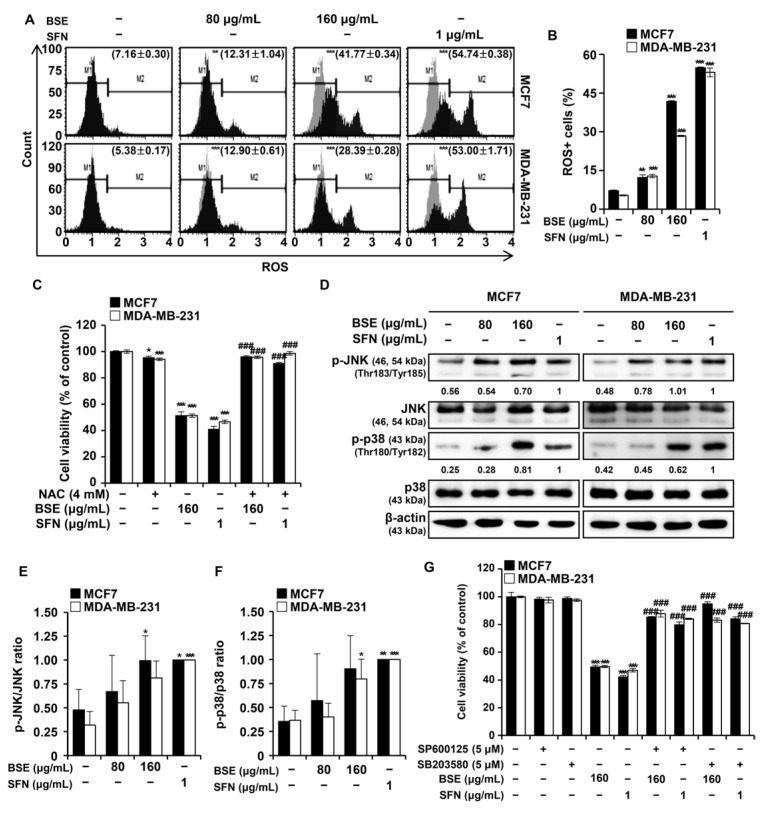
Broccoli sprout extract (BSE) or sulforaphane (SFN) induced reactive oxygen species (ROS) generation and activated the p38/c-Jun N-terminal kinase (JNK) signaling pathway in breast cancer cells. (**A**) MCF7 and MDA-MB-231 cells were treated with the indicated concentrations of BSE or SFN for 48 h, followed by incubation with 5 μM CellROX Green Reagent (C10444; Invitrogen, Carlsbad, CA, USA) for 30 min at 37 °C. (**B**) Quantitative analysis of ROS generation is shown in histograms. ROS levels were measured using a MACSQuant Analyzer. (**C**) Cells were pretreated with N-acetylcysteine (NAC) for 3 h and subsequently treated with the indicated concentrations of BSE or SFN; the effects of BSE and SFN were abrogated in the presence of NAC. (**D**) Expression of p38, phosphorylated p38 (p-p38), JNK, and phosphorylated JNK (p-JNK) was assessed by Western blot. The numbers below the bands represent the relative protein expression levels normalized to β-actin, as determined by densitometric analysis. (**E**,**F**) Ratios of phosphorylated to total protein for JNK and p38 are presented. (**G**) Cell viability was measured by the MTT assay after 48 h of treatment with BSE or SFN, the JNK inhibitor SP600125, and the p38 inhibitor SB203580. Data are shown as mean ± standard deviation (*n* = 3). (**B**,**E**,**F**) One-way ANOVA followed by Tukey’s post hoc test; (**C**,**G**) Unpaired t-tests vs. BSE or SFN alone. * *p* < 0.05, ** *p* < 0.01, and *** *p* < 0.001 versus vehicle-treated cells; ^###^ *p* < 0.001 versus BSE- or SFN-only treated cells.

ROS are byproducts of cellular metabolism that are generated primarily in mitochondria and play critical roles in cell development, metabolic regulation, intracellular signaling, and mitochondrial function [21]. Flow cytometric analysis using an oxidative stress detection kit demonstrated that treatment with BSE or SFN increased intracellular ROS levels in MCF7 and MDA-MB-231 breast cancer cells (Figure 2A). The proportion of cells exhibiting elevated ROS levels is presented in histogram form (Figure 2B). To determine whether ROS generation was required for the cytotoxic effects of BSE and SFN, cells were co-treated with the ROS scavenger N-acetylcysteine (NAC). NAC co-treatment significantly restored cell viability compared with treatment with BSE or SFN alone, whereas NAC treatment alone did not significantly affect cell viability (Figure 2C). Western blot analysis demonstrated increased phosphorylation of JNK and p38 following treatment with BSE or SFN, whereas total JNK and p38 protein levels remained relatively unchanged (Figure 2D). Densitometric analyses of the ratios of phosphorylated to total protein are presented in Figure 2E,F. To investigate the roles of these kinases, breast cancer cells were pretreated with SP600125, a JNK inhibitor, or SB203580, a p38 inhibitor, before exposure to BSE or SFN. Cell viability, assessed by the MTT assay, was restored significantly in the presence of either inhibitor, indicating that activation of JNK and p38 played critical roles in BSE- or SFN-induced apoptotic progression (Figure 2G).

### 3.4. BSE and SFN Induce G2/M Phase Arrest in MCF7 and MDA MB-231 Cells

**Figure 3:**
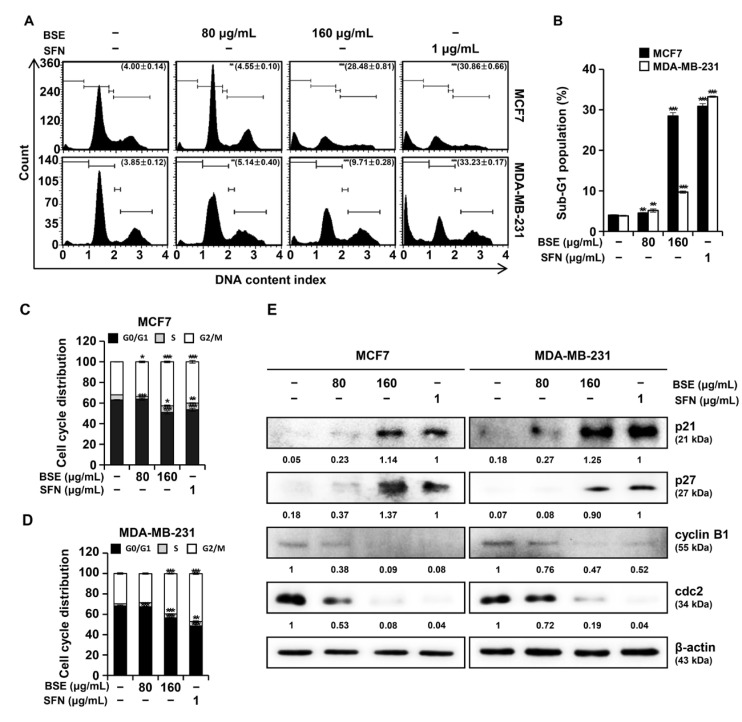
Broccoli sprout extract (BSE) and sulforaphane (SFN) induced G2/M phase cell cycle arrest in MCF7 and MDA-MB-231 cells. MCF7 and MDA-MB-231 cells were treated with BSE (80 and 160 μg/mL) or SFN (1 μg/mL) for 48 h. (**A**) Cell cycle analysis was performed by flow cytometry using propidium iodide staining. (**B**) Percentage of cells in the sub-G1 phase. (**C**,**D**) Distribution of cells across cell cycle phases. Data are presented as mean ± standard deviation from three independent experiments. One-way ANOVA followed by Tukey’s post hoc test; * *p* < 0.05, ** *p* < 0.01, *** *p* < 0.001 versus vehicle-treated cells. (**E**) Western blot analysis of cell cycle–related proteins, including p21, p27, cyclin B1, and cdc2, with β-actin as a loading control. The numbers below the bands represent the relative protein expression levels normalized to β-actin, as determined by densitometric analysis.

To evaluate the effects of BSE and SFN on cell cycle distribution, MCF7 and MDA-MB-231 cells were analyzed using flow cytometry after PI staining (Figure 3A). In MCF7 cells, the respective Sub-G1 populations increased from 4.00% to 4.55%, 28.48%, and 30.86% following treatment with BSE (80 μg/mL, 160 μg/mL) and SFN (1 μg/mL). Similarly, in MDA-MB-231 cells, the Sub-G1 fractions increased from 3.85% to 5.14%, 9.71%, and 33.23%, respectively, indicating apoptosis induction by these compounds (Figure 3B). Concomitantly, the respective proportions of MCF7 cells in the G2/M phase increased from 32.03% to 33.69%, 42.63%, and 40.09%, whereas in MDA-MB-231 cells, they increased from 29.69% to 28.49%, 39.54%, and 46.92%, respectively (Figure 3C,D), demonstrating G2/M phase arrest. Western blot analysis revealed that treatment with BSE or SFN upregulated the CDK inhibitors p21 and p27, while reducing cyclin B1 and cdc2 levels in both cell lines, supporting the observed cell cycle arrest (Figure 3E). The observed G2/M phase arrest was closely associated with a concomitant increase in the Sub-G1 population, which serves as a marker for fragmented DNA during late-stage apoptosis. These results, when cross-referenced with the Annexin V/7-AAD staining data (Figure 1D), confirm that the arrest of breast cancer cells at the mitotic checkpoint significantly contributes to the overall induction of programmed cell death by BSE and SFN.

### 3.5. BSE and SFN Induce Endoplasmic Reticulum (ER) Stress and Loss of MMP in the Breast Cancer Cells

**Figure 4:**
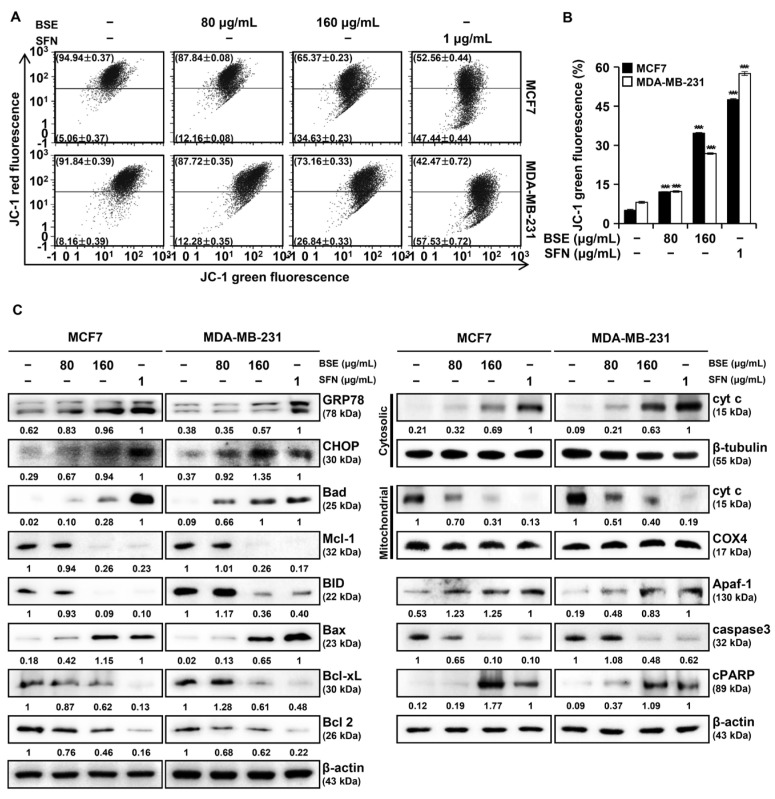
Broccoli sprout extract (BSE) and sulforaphane (SFN) activated the mitochondrial apoptotic pathway in MCF7 and MDA-MB-231 cells. (**A**,**B**) JC-1 staining was used to assess mitochondrial membrane potential; BSE or SFN treatment disrupted mitochondrial membrane potential in MCF7 and MDA-MB-231 cells. Data are presented as mean ± SD (*n* = 3). One-way ANOVA followed by Tukey’s post hoc test; *** *p* < 0.001 versus vehicle-treated cells. (**C**) The effects of BSE or SFN on proteins associated with the mitochondrial apoptotic pathway were analyzed by Western blot. The proteins assessed included glucose-regulated protein 78 (GRP78), C/EBP homologous protein (CHOP), BCL2-associated agonist of cell death (Bad), myeloid cell leukemia-1 (Mcl-1), BH3-interacting domain death agonist (BID), BCL2-associated X pro-tein (Bax), B-cell lymphoma-extra-large (Bcl-xL), B-cell lymphoma 2 (Bcl 2), cytochrome c (cyt c), β-tubulin, cytochrome c oxidase subunit 4 (COX4), apoptotic protease activating factor-1 (Apaf-1), caspase3, and cleaved poly(ADP-ribose) polymerase (cPARP). β-tubulin (cytosolic marker), COX4 (mitochondrial marker). β-actin served as the loading control. The numbers below the bands represent the relative protein expression levels normalized to β-actin, as determined by densitometric analysis.

Mitochondrial dysfunction is a hallmark of apoptosis, with loss of MMP serving as an indicator of mitochondrial damage [22]. To evaluate the effects of BSE and SFN on mitochondrial integrity, MCF7 and MDA-MB-231 cells were treated with BSE or SFN and analyzed by flow cytometry using JC-1 staining. Dot plots and histograms revealed increased green fluorescence, indicating MMP disruption following treatment (Figure 4A,B). To assess cyt c translocation, mitochondrial and cytosolic fractions were isolated and analyzed by Western blot; in addition, whole-cell lysates were examined for Bcl-2 family and other apoptosis-related proteins. BSE or SFN treatment decreased mitochondrial cyt c levels while significantly increasing its cytosolic fraction (Figure 4C). CHOP, a central mediator of ER stress-induced apoptosis, was upregulated. The pro-apoptotic proteins Bad and Bax were increased, whereas the anti-apoptotic proteins BID, Bcl 2, and Bcl-xL were downregulated. These alterations were accompanied by elevated Apaf-1 expression and caspase-3 cleavage, indicating activation of the apoptotic cascade.

### 3.6. BSE and SFN Enhanced Multi-Caspase Activity in Breast Cancer Cells

**Figure 5:**
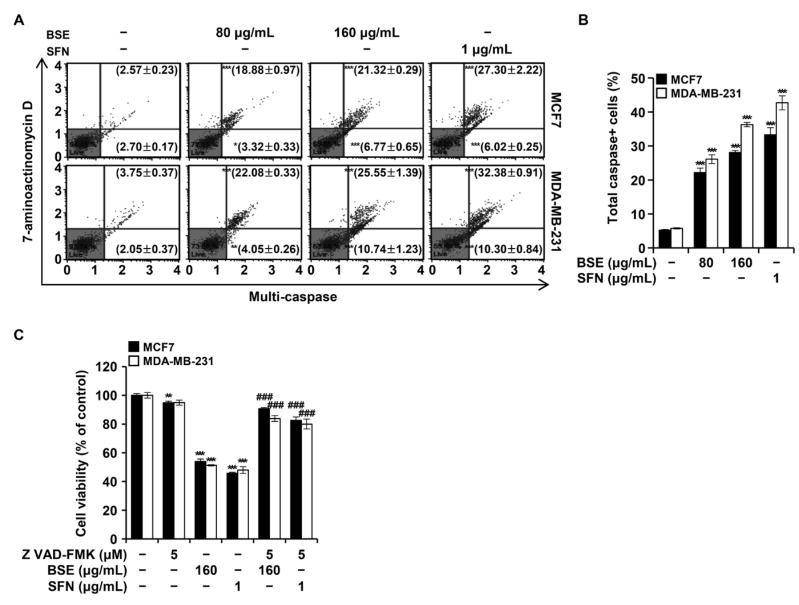
Broccoli sprout extract (BSE) and sulforaphane (SFN) activated caspases in MCF7 and MDA-MB-231 cells. MCF7 and MDA-MB-231 cells were treated with BSE (80 and 160 μg/mL) or SFN (1 μg/mL) for 48 h. (**A**) Caspase activation was analyzed by flow cytometry using a Muse Multi-Caspase Kit. (**B**) The percentage of cells with activated caspases was calculated by summing cells in the upper right quadrant (caspase-positive, non-viable) and lower right quadrant (caspase-positive, viable). (**C**) Cell viability was assessed by the MTT assay after 48 h treatment with BSE, SFN, or the pan-caspase inhibitor Z-VAD-FMK as indicated. Data are presented as mean ± SD (*n* = 3). Unpaired t-tests vs. BSE or SFN alone. * *p* < 0.05, ** *p* < 0.01, *** *p* < 0.001 versus vehicle-treated cells; ^###^ *p* < 0.001 versus BSE- or SFN-only treated cells.

During apoptosis, caspases, which are cysteine-dependent aspartate-specific proteases, mediate DNA degradation, alter cell morphology, and drive cell death [23]. To evaluate multi-caspase activation by BSE and SFN, MCF7 and MDA-MB-231 cells were treated with the indicated concentrations of each compound for 48 h (Figure 5A,B). In MCF7 cells, the proportions of cells with activated caspases increased significantly from 5.27% in controls to 22.20%, 28.08%, and 33.32% following treatment with BSE (80 μg/mL, 160 μg/mL) and SFN (1 μg/mL), respectively. Similarly, in MDA-MB-231 cells, caspase-positive cells increased from 5.80% to 26.13%, 36.30%, and 42.68%, respectively. To determine whether caspase activation is essential for BSE- or SFN-induced apoptosis, the cells were co-treated with Z-VAD-FMK, a broad-spectrum caspase inhibitor (Figure 5C). In MCF7 cells, viability, which had declined to 54.02% and 45.69% after BSE (160 μg/mL) and SFN (1 μg/mL) treatment, respectively, recovered to 90.82% and 82.65%, respectively, with Z-VAD-FMK co-treatment. Similarly, MDA-MB-231 cell viability, which had decreased to 51.22% and 47.90%, respectively, recovered to 83.88% and 80.03%, respectively. These findings indicate that BSE- and SFN-induced apoptosis in breast cancer cells is largely dependent on caspase activation.

### 3.7. In Vivo Oral Absorption of SFN and SFN-Containing BSE in Rats

**Figure 6:**
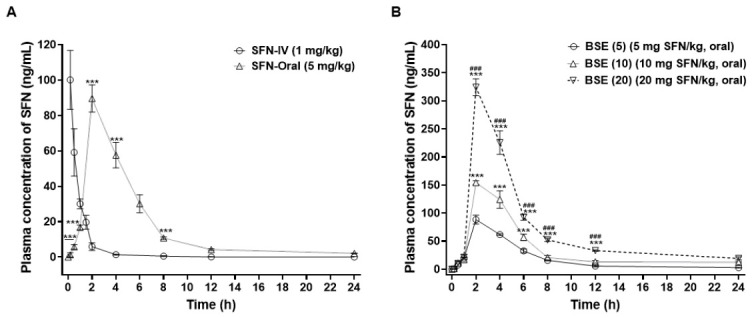
Broccoli sprout extract (BSE) facilitated sustained and dose-dependent exposure of sulforaphane (SFN) *in vivo*. Male Sprague-Dawley rats were administered SFN intravenously (SFN-IV, 1 mg/kg) or orally (SFN-Oral, 5 mg/kg), or orally with BSE containing SFN at equivalent doses of 5, 10, or 20 mg SFN/kg. Plasma samples were collected at designated time points, and SFN concentrations were quantified using ultra-performance liquid chromatography–tandem mass spectrometry (UPLC–MS/MS). (**A**) Plasma concentration–time profiles of SFN following intravenous (SFN-IV, 1 mg/kg) and oral (SFN-Oral, 5 mg/kg) administration. Data are presented as mean ± SD (*n* = 3 per group). Statistical analysis was performed using a paired Student’s t-test. *** *p* < 0.001 versus SFN-IV (1 mg/kg). (**B**) Plasma concentration–time profiles of SFN following oral administration of BSE at SFN-equivalent doses of 5, 10, and 20 mg/kg. Data are presented as mean ± SD (*n* = 3 per group). Statistical analysis was performed using one-way ANOVA followed by Tukey’s post hoc test. *** *p* < 0.001 versus BSE (5); ^###^ *p* < 0.001 versus BSE (10).

Systemic exposure to SFN following oral administration was evaluated in male Sprague–Dawley rats using pure SFN and BSE at SFN-equivalent doses of 5, 10, and 20 mg/kg. An IV SFN group (1 mg/kg) was included to determine absolute oral bioavailability (Figure 6A,B). Following IV administration of SFN (1 mg/kg), plasma concentration–time profiles demonstrated relatively rapid elimination, with a terminal half-life (T_1/2_) of 1.48 ± 0.93 h and a maximum concentration (C_max_) of 100 ± 16.7 ng/mL. The corresponding areas under the curve (AUC_last_ and AUC_inf_) were 99.1 ± 7.38 and 99.7 ± 7.80 ng·h/mL, respectively. Oral administration of pure SFN at 5 mg/kg resulted in a gradual increase in plasma levels, reaching a peak concentration of 89.6 ± 7.66 ng/mL at 2 h. The AUC_last_ was 406 ± 31.1 ng·h/mL, corresponding to an oral bioavailability of 82.0 ± 6.28%. Administration of BSE at an equivalent SFN dose (5 mg/kg) produced a comparable peak plasma concentration (88.5 ± 7.92 ng/mL), systemic exposure (AUC_last_ 445 ± 5.16 ng·h/mL), and bioavailability (89.9 ± 1.04%) to those of orally administered pure SFN. Although the BSE group showed a trend toward higher systemic exposure compared with SFN, the differences were not statistically significant (*p* > 0.05).

Furthermore, escalation of the oral SFN-equivalent dose to 10 and 20 mg/kg resulted in peak plasma concentrations of 154 ± 3.39 ng/mL and 324 ± 15.0 ng/mL, respectively. The T_1/2_ of SFN was prolonged to 9.11 ± 5.65 h and 10.5 ± 3.02 h at 10 and 20 mg/kg, respectively, leading to increased overall systemic exposure, with AUC_last_ values of 848 ± 13.0 ng·h/mL and 1664 ± 27.6 ng·h/mL and corresponding oral bioavailability of 85.6 ± 1.32% and 83.7 ± 1.39%. The observed variability in pharmacokinetic parameters (e.g., C_max_ and AUC) is reflected in the reported SD values and is consistent with expected inter-individual variation in *in vivo* pharmacokinetic studies. Statistical comparisons indicated no significant difference between BSE and pure SFN at the equivalent dose (5 mg/kg), while dose escalation resulted in significant increases in systemic exposure within the BSE groups. Collectively, oral administration of BSE resulted in sustained and dose-dependent systemic exposure to SFN.

### 3.8. BSE Exerts Antitumor Activity in a Xenograft Mouse Model

**Figure 7:**
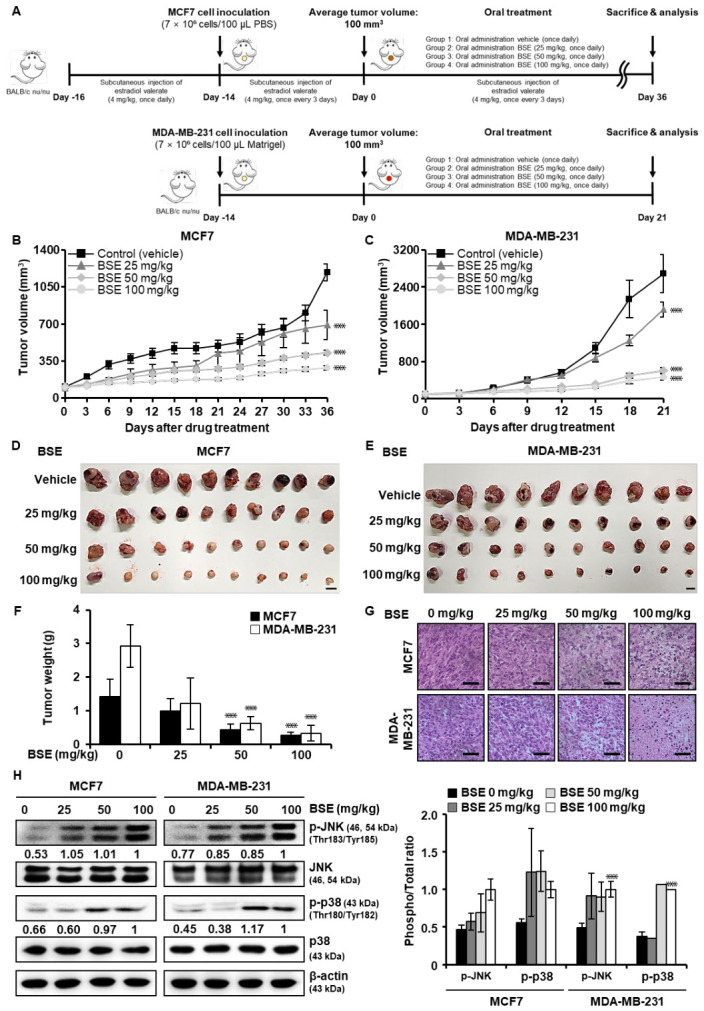

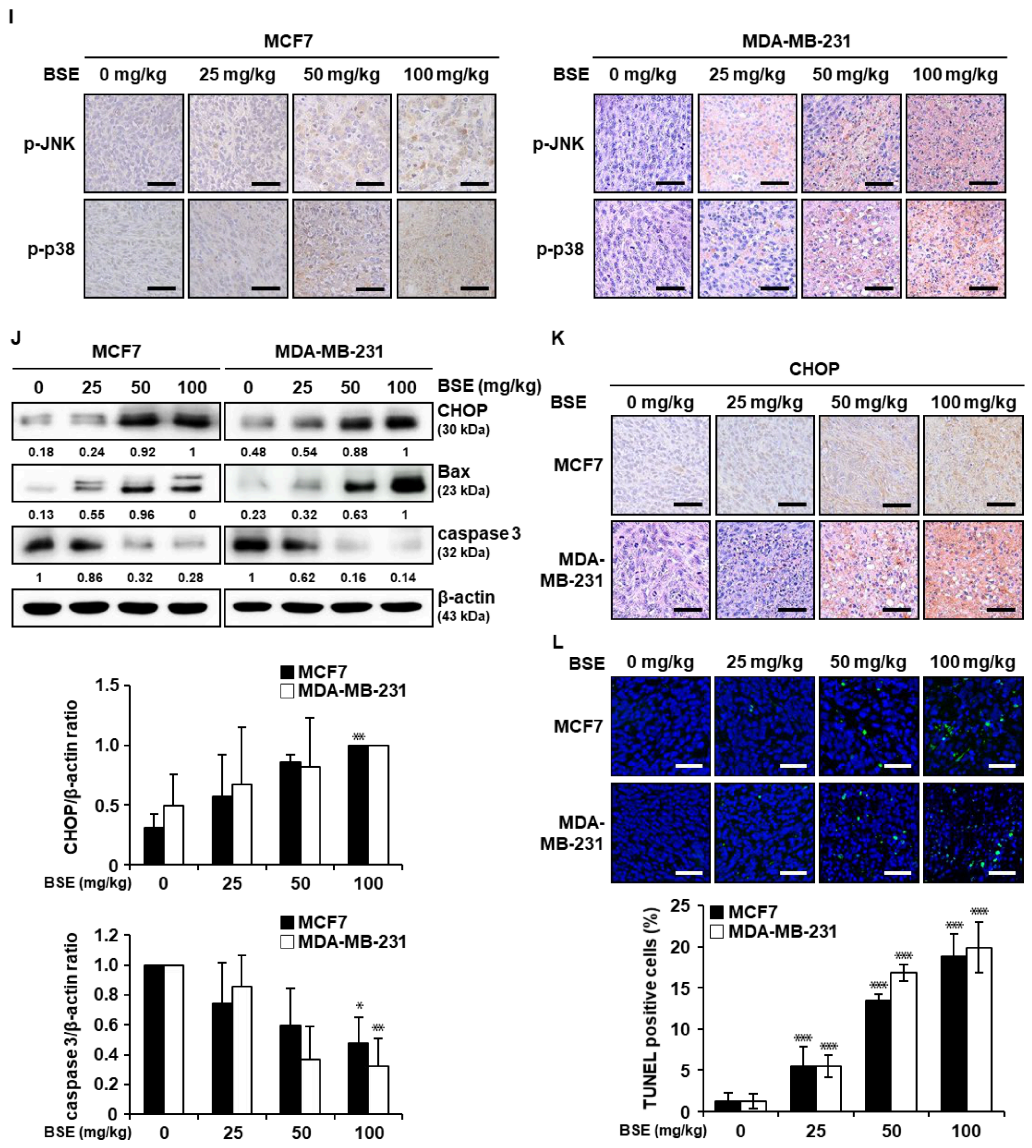
Oral administration of broccoli sprout extract (BSE) inhibited tumor growth in MCF7 and MDA-MB-231 xenograft mouse models. (**A**) Female BALB/c nude mice were inoculated with MCF7 or MDA-MB-231 cells and treated orally with BSE (0, 25, 50, or 100 mg/kg) for up to 36 days (MCF7) or 21 days (MDA-MB-231). (**B**,**C**) Tumor growth curves of MCF7 and MDA-MB-231 xenografts during the treatment period (MCF7: 36 days; MDA-MB-231: 21 days). Data are presented as mean ± SD (*n* = 10 per group). (**D**,**E**) Representative images of excised tumors from MCF7 and MDA-MB-231 xenografts (scale bar, 10 mm). (**F**) Tumor weights shown as individual data point overlays. (**G**) Representative hematoxylin and eosin (H&E) staining of tumor sections (scale bar, 50 µm). Data are presented as mean ± SD (*n* = 10 per group). (**H**) The effects of BSE on stress-activated mitogen-activated protein kinase (MAPK) signaling in tumor lysates were analyzed by Western blot. Protein levels of phosphorylated c-Jun N-terminal kinase (p-JNK), total JNK, phosphorylated p38 (p-p38), and total p38 were assessed, with β-actin used as a loading control. Quantification of p-JNK/JNK and p-p38/p38 ratios is shown. Data are presented as mean ± SD (*n* = 3 per group). The numbers below the bands represent the relative protein expression levels normalized to β-actin, as determined by densitometric analysis. (**I**) Representative immunohistochemistry images of p-JNK and p-p38 in tumor sections from MCF7 and MDA-MB-231 xenografts (scale bar, 50 µm). (**J**) The effects of BSE on apoptosis-related proteins in tumor lysates were assessed by Western blot. Protein levels of C/EBP homologous protein (CHOP), BCL2-associated X protein (Bax), and caspase3 were analyzed, with β-actin used as a loading control. The numbers below the bands represent the relative protein expression levels normalized to β-actin, as determined by densitometric analysis. Quantification of CHOP/β-actin and caspase3/β-actin ratios is shown (mean ± SD, *n* = 3 per group). (**K**) Representative immunohistochemistry images of CHOP in tumor sections (scale bar, 50 µm). (**L**) Representative terminal deoxynucleotidyl transferase dUTP nick end labeling (TUNEL) assay images of tumor sections (upper) and quantification of TUNEL-positive cells (lower) (scale bar, 50 µm). Statistical analysis was performed using one-way ANOVA followed by Tukey’s post hoc test. * *p* < 0.05, ** *p* < 0.01, *** *p* < 0.001 versus vehicle-treated cells. All panels include MCF7 and MDA-MB-231 xenograft models.

To assess whether the potent antitumor effects of BSE observed *in vitro* could be reproduced *in vivo*, we evaluated oral BSE administration in MCF7 and MDA-MB-231 human breast cancer xenograft models (Figure 7A). Oral BSE treatment inhibited tumor growth in a dose-dependent manner in both models. In contrast, vehicle-treated control mice exhibited rapid tumor progression, whereas BSE-treated groups exhibited marked attenuation of tumor growth, with the 100 mg/kg dose consistently producing the most pronounced antitumor effect among the tested doses (Figure 7B,C). During the early treatment period (days 3–9), tumor volumes increased from approximately 200 to 371 mm^3^ in MCF7 controls and from 122 to 387 mm^3^ in MDA-MB-231 controls. In contrast, BSE at 25, 50, or 100 mg/kg substantially suppressed tumor growth. By the mid-to-late treatment period, the divergence between control and BSE-treated groups became more pronounced. At representative mid-to-late treatment time points, tumor volumes were reduced relative to control by approximately 18.1%, 49.5%, and 66.1% in MCF7 xenografts on day 33, and by approximately 41.7%, 77.0%, and 82.2% in MDA-MB-231 xenografts on day 18, at 25, 50, and 100 mg/kg, respectively. At the final treatment points, tumor volumes were significantly smaller in BSE-treated groups than in controls, with reductions of approximately 42.0%, 64.0%, and 76.1% in MCF7 xenografts on day 36, and 28.5%, 77.4%, and 82.6% in MDA-MB-231 xenografts on day 21, at 25, 50, and 100 mg/kg, respectively (Figure 7D,E). These results indicate a consistent and dose-dependent suppression of tumor growth across both xenograft models.

Consistent with tumor volume measurements, excised tumor weights exhibited dose-dependent reductions. In control animals, mean tumor weights were 1.42 g in MCF7 xenografts and 2.92 g in MDA-MB-231 xenografts. In MCF7 xenografts, BSE treatment at 25, 50, and 100 mg/kg reduced mean tumor weights to 1.00, 0.44, and 0.26 g, respectively, representing approximately 1.42-, 3.23-, and 5.46-fold reductions relative to control. In MDA-MB-231 xenografts, the corresponding mean tumor weights were 1.21, 0.63, and 0.33 g, representing approximately 2.41-, 4.63-, and 8.85-fold reductions, respectively (Figure 7F). These findings demonstrate that oral BSE significantly inhibited tumor growth in a dose-dependent manner in both xenograft models without overt signs of systemic toxicity.

To evaluate histopathological changes following BSE treatment, H&E staining was performed on excised tumor tissues. Histological analysis revealed dose-dependent alterations in tumor architecture in both MCF7 and MDA-MB-231 xenografts. Tumors from vehicle-treated mice in both models exhibited dense cellularity, frequent mitotic figures, and minimal necrotic areas. In contrast, particularly at doses of 50 and 100 mg/kg, BSE-treated tumors exhibited reduced cellular density, prominent cytoplasmic vacuolization, and expanded regions of necrosis (Figure 7G). These histopathological features were observed consistently across breast cancer models and correlated with the reductions in tumor volume and weight, supporting the dose-dependent antitumor efficacy of BSE.

To determine whether the antitumor effects of BSE observed *in vivo* were consistent with mechanisms identified *in vitro* (Figure 2 and Figure 4), tumor tissues from MCF7 and MDA-MB-231 xenografts were analyzed by Western blotting and immunohistochemistry. Based on the *in vitro* findings indicating that BSE induced ROS–mediated activation of MAPK signaling, JNK phosphorylation and p38 phosphorylation were selected as representative markers for *in vivo* validation, as these proteins are central components of stress-activated MAPK pathways. Western blot analysis demonstrated dose-dependent increases in JNK and p38 phosphorylation in tumor tissues from BSE-treated mice, whereas total JNK and p38 protein levels remained relatively unchanged (Figure 7H). Consistently, immunohistochemical staining revealed dose-dependent increases in JNK phosphorylation and p38 phosphorylation in tumors from BSE-treated mice compared to vehicle-treated controls in MCF7 and MDA-MB-231 xenografts (Figure 7I). These findings demonstrate that BSE induced dose-dependent activation of stress-activated MAPK pathways and endoplasmic reticulum stress in tumor tissues *in vivo*.

Considering that BSE-induced ER stress-mediated apoptosis was demonstrated *in vitro*, tumor tissues from xenograft models were analyzed to confirm the expression of ER stress–related apoptotic markers *in vivo*. Western blot analysis revealed dose-dependent increases in CHOP, Bax, and cleaved caspase 3 following BSE treatment (Figure 7J). Among these markers, CHOP was further evaluated by immunohistochemistry as a representative ER stress effector, and CHOP-positive staining was found to be significantly increased in tumors from BSE-treated mice (Figure 7K). To assess apoptotic cell death *in vivo* directly, TUNEL assays were performed on tumor tissues. A dose-dependent increase in TUNEL-positive cells was observed in BSE-treated tumors compared to vehicle-treated controls. In the MCF7 xenograft model, the proportion of TUNEL-positive cells increased from 1.17% in control tumors to 5.46%, 13.48%, and 18.88% following BSE treatment at 25, 50, and 100 mg/kg, respectively. Similarly, in the MDA-MB-231 model, TUNEL-positive cells increased from 1.19% in controls to 5.44%, 16.82%, and 19.89%, respectively (Figure 7L). These findings demonstrate enhanced apoptotic cell death *in vivo* and further support that BSE induces apoptosis in hormone-dependent and TNBC xenograft models, consistent with the mechanistic pathways identified *in vitro*.

After 5 weeks of metronomic oral administration of BSE, serum biochemical parameters were evaluated to assess hepatic function, renal status, and electrolyte balance (Figure S1). Across all tested doses, hepatic enzyme levels remained within physiological ranges. At the selected optimal dose of BSE (100 mg/kg), aspartate aminotransferase, alanine aminotransferase, alkaline phosphatase, and total bilirubin were 95.0 ± 2.79 U/L, 46.5 ± 1.58 U/L, 141 ± 3.82 U/L, and 0.433 ± 0.02 mg/dL, respectively, closely approximating the values observed in untreated animals. Renal function markers and electrolyte parameters were likewise maintained within expected ranges following BSE administration. Blood urea nitrogen levels ranged from 21.7 ± 1.49 to 24.8 ± 1.32 mg/dL, and serum creatinine values ranged from 0.327 ± 0.015 to 0.389 ± 0.013 mg/dL (Figure S1). In addition, electrolyte concentrations, including phosphorus and calcium, were within the ranges of 5.30 ± 0.12 to 5.80 ± 0.12 mg/dL and 9.40 ± 0.12 to 9.69 ± 0.12 mg/dL, respectively, indicating the absence of overt renal toxicity at the evaluated doses.

Consistent with the biochemical findings, histopathological examination of major organs—including the liver, kidney, intestine, spleen, lung, and heart—demonstrated preserved tissue architecture across all oral BSE dose groups (Figure S2). No structural abnormalities, inflammatory infiltrates, or degenerative changes were detected in the examined tissues, supporting the absence of severe systemic toxicity following prolonged oral BSE administration.

Collectively, oral BSE administration effectively inhibited tumor growth, reduced tumor weight, and induced apoptosis in MCF7 and MDA-MB-231 xenograft models. These antitumor effects were associated with dose-dependent activation of stress-activated MAPK signaling, ER stress, and downstream apoptotic pathways, consistent with the mechanisms identified *in vitro*. These findings support BSE as a safe and effective oral anticancer agent.

## 4. Discussion

Breast cancer remains a major global health burden, underscoring the need for improved therapeutic strategies to overcome treatment resistance and enhance patient outcomes. In this study, BSE and its bioactive component, SFN, exhibited significant anticancer effects in both MCF7 cells, a hormone-dependent breast cancer model, and MDA-MB-231 cells representing TNBC [24], through modulation of multiple oncogenic pathways. Cell viability and colony formation assays demonstrated that both BSE and SFN inhibited breast cancer cell growth by inducing apoptosis, as confirmed by Annexin V staining (Figure 1). The calculated IC_50_ values further suggest that, on a nominal concentration basis, SFN accounts for only a small fraction of the overall cytotoxic potency of BSE, assuming no synergistic or antagonistic interactions among coexisting constituents. This observation supports the possibility that the anticancer activity of BSE may not be attributable to SFN alone, but may also be influenced by other bioactive components present within the extract, which warrants further investigation.

ROS play a dual role in cancer progression, with moderate levels supporting tumor cell survival and excessive accumulation inducing oxidative damage that culminates in apoptosis [25]. In this context, the increase in ROS observed following BSE and SFN treatment may be associated with activation of apoptotic signaling pathways in breast cancer cells. In our study, BSE or SFN treatment induced a significant increase in intracellular ROS levels in MCF7 and MDA-MB-231 cells, which was associated with reduced cell viability (Figure 2A,B). Pretreatment with NAC, a ROS scavenger, significantly attenuated the cytotoxic effects of BSE and SFN, supporting an important role for oxidative stress in mediating their anticancer activity (Figure 2C). While the attenuation of cytotoxicity by NAC highlights the involvement of ROS, we acknowledge that relying solely on a pharmacological scavenger has limitations in definitively establishing ROS dependency [26]. Future studies incorporating genetic approaches, such as the targeted knockdown of specific antioxidant systems, are warranted to confirm these precise ROS-dependent molecular mechanisms. Consistent with ROS-mediated stress signaling, treatment with BSE or SFN resulted in activation of JNK and p38 MAPK, which are key regulators of stress-induced apoptosis (Figure 2D–F) [27]. The functional importance of these pathways was further confirmed using selective pharmacological inhibitors, as pretreatment with SP600125 (JNK inhibitor) or SB203580 (p38 inhibitor) significantly abrogated BSE- and SFN-induced cytotoxicity (Figure 2G). Although our data highlight the ROS/MAPK axis as a significant mediator of BSE-induced apoptosis, the pleiotropic nature of phytochemicals suggests the involvement of additional signaling networks. ROS generation can simultaneously trigger alternative cell death cascades and suppress oncogenic survival pathways, such as PI3K/Akt and Wnt/beta-catenin signaling, or induce lipid peroxidation-dependent ferroptosis [28]. Future functional validation using genetic approaches, such as siRNA-mediated knock-down of JNK or p38, is warranted to complement our current pharmacological findings.

In addition, our findings reveal that BSE and SFN induced G2/M phase cell cycle arrest in both MCF7 and MDA-MB-231 cells (Figure 3A,B). The increased accumulation of cells in the G2/M phase (Figure 3C,D) indicates disruption of normal cell cycle progression. Consistent with these findings, Western blot analysis demonstrated upregulation of the cyclin-dependent kinase inhibitors p21 and p27, accompanied by downregulation of cyclin B1 and cdc2, which are critical regulators of mitotic entry (Figure 3E). Mechanistically, the enforced G2/M arrest serves as a regulatory gateway; by inhibiting the cyclin B1/cdc2 complex through the upregulation of p21 and p27, BSE prevents damaged cells from undergoing mitosis. This sustained cell cycle blockade likely acts as a functional precursor to apoptosis, shifting the cellular balance toward the intrinsic death pathway as evidenced by the subsequent mitochondrial dysfunction and caspase activation [29,30]. These findings indicate that BSE and SFN suppressed breast cancer cell proliferation by enforcing cell cycle arrest at the G2/M checkpoint.

ROS are key mediators of ER stress and modulate the balance of Bcl-2 family proteins, leading to mitochondrial membrane dysfunction [31]. Consistent with these mechanisms, loss of MMP was observed in MCF7 and MDA-MB-231 cells following treatment with BSE or SFN (Figure 4A,B). Mcl-1, frequently overexpressed in cancers, serves as a primary survival factor; thus, its suppression by BSE represents a critical mechanism for bypassing apoptotic resistance [32]. Furthermore, the activation of BID suggests a potent crosstalk where BSE-induced ROS and subsequent MAPK signaling may promote BID cleavage to amplify mitochondrial death signals [33]. The observed upregulation of the proapoptotic proteins Bax and BID, together with downregulation of the antiapoptotic proteins Bcl 2 and Bcl-xL, indicates that BSE and SFN induce apoptosis predominantly through the intrinsic mitochondrial pathway, as further supported by cyt c release and cleavage of caspase-3 and PARP (Figure 4C) [34]. Caspase activation was a prominent feature of BSE- and SFN-induced apoptosis. The significant increase in multi-caspase activity detected using flow cytometry implies involvement of extrinsic and intrinsic apoptotic pathways (Figure 5A,B). Moreover, the partial restoration of cell viability by the pan-caspase inhibitor Z-VAD-FMK confirmed that apoptosis was a principal mechanism of BSE- and SFN-induced cell death (Figure 5C).

The 37 °C incubation step used during BSE preparation was intended to enhance SFN formation prior to extraction. Based on our previous work, this conversion is most plausibly explained by the action of endogenous myrosinase released following plant tissue disruption, and optimization of incubation conditions was associated with increased SFN yield [16]. In the present study, because myrosinase activity was not directly quantified after drying, pulverization, or storage, the extent of enzyme preservation during preprocessing cannot be defined quantitatively. Nevertheless, the substantial SFN content detected in the final extract suggests that sufficient endogenous conversion capacity was retained under the mild pretreatment conditions employed.

Pharmacokinetic analysis in rats demonstrated that BSE enables sustained systemic exposure to SFN, characterized by comparable C_max_ values and moderately increased AUC_last_ and oral bioavailability at an equivalent SFN dose of 5 mg/kg. These findings imply that incorporation of SFN within the BSE matrix facilitates efficient oral absorption. Although relative oral bioavailability exhibited a modest but consistent decline with increasing BSE dose both C_max_ and AUC_last_ increased proportionally, indicating clear dose-dependent enhancement of overall systemic exposure. Notably, prolongation of the apparent T_1/2_ was observed at higher doses, a phenomenon more plausibly attributed to absorption-related processes rather than alterations in intrinsic elimination. This pharmacokinetic profile is consistent with flip-flop kinetics, in which sustained gastrointestinal input governs the terminal phase [34]. In addition, the high fiber content of BSE may contribute to delayed gastrointestinal transit and prolonged exposure of SFN to absorptive regions of the intestine. Although these interpretations are plausible, further mechanistic studies are warranted to delineate the specific absorption pathways involved.

The rat pharmacokinetic study was designed to provide exposure-supportive information for oral SFN delivery by BSE and to support dose selection for the xenograft study, rather than to establish a species- and sex-matched PK/PD relationship with the efficacy model.

The rat pharmacokinetic findings, particularly the sustained systemic exposure achieved after oral BSE administration, provide supportive evidence for evaluating BSE as an oral SFN-containing formulation in the xenograft model; however, these findings should not be interpreted as a direct species-matched predictor of tumor response. Notably, the dose range used in this study represents a relatively low SFN-equivalent exposure level compared with previously reported high-dose regimens, suggesting that the observed efficacy may be associated with sustained systemic exposure under repeated oral administration conditions [18,19,20].

*In vivo* efficacy was further confirmed in xenograft mouse models, in which oral administration of BSE significantly suppressed tumor growth in MCF7 and MDA-MB-231 breast cancer models in a dose-dependent manner. Tumor inhibition was maintained throughout the treatment period and was consistent with the antiproliferative effects observed *in vitro*. Western blot and immunohistochemical analyses further showed that BSE treatment was associated with activation of stress-related MAPK signaling and ER stress-associated apoptotic pathways, as evidenced by increased levels of p-JNK, p-p38, CHOP, Bax, and cleaved caspase 3 [34].

Repeated metronomic oral administration of BSE was well tolerated across all tested doses [34]. Serum biochemical parameters reflecting hepatic and renal function, as well as electrolyte balance, remained within physiological ranges; these findings were corroborated by histopathological examination, which revealed no evidence of inflammatory or degenerative changes in major organs. Collectively, these results indicate a favorable systemic safety profile for repeated oral administration of BSE under the tested conditions.

Importantly, BSE is not a single-compound preparation but a chemically complex phytochemical mixture. Previous studies, including our own, have demonstrated that BSE contains diverse classes of bioactive constituents identified through LC-MS/MS-based chemical profiling and molecular networking analyses. Specifically, the extract is composed predominantly of amino acids (~23%) and alkaloids (~20%), followed by organic acids (~5%), fatty acids and glycosides (~4% each), as well as terpenes and sesquiterpenoids (~3% each). In addition to SFN, glucosinolate-derived isothiocyanates and precursor compounds such as glucoraphanin are also present within the extract matrix. Major identified components include arginine, proline, phenylalanine, adenine, adenosine, and SFN, among others [18].

This compositional complexity suggests that the biological activity of BSE may not be attributable solely to SFN, but -may also involve contributions from coexisting constituents. Indeed, BSE has been reported to exhibit enhanced antioxidant capacity compared with pure SFN, supporting the possibility of synergistic or additive effects among multiple components. In the context of the present study, such interactions may influence both the pharmacokinetic behavior of SFN and its downstream biological effects, including systemic exposure and anticancer activity.

Although SFN is considered the principal marker compound, the contribution of other constituents cannot be excluded. Therefore, the observed anticancer effects of BSE should be interpreted as arising from a complex phytochemical system, and further studies are warranted to quantitatively define the roles and interactions of individual components.

The ability of BSE to suppress tumor growth across molecularly distinct breast cancer subtypes implies a mechanism of action that is independent of hormone receptor status, underscoring its potential applicability in heterogeneous disease settings. Notably, BSE suppressed tumor enlargement from the early stages of tumor development and maintained sustained antitumor efficacy throughout the treatment period in hormone-dependent MCF7 and triple-negative MDA MB-231 models. As TNBC lacks targetable receptors and is refractory to conventional chemotherapeutic and endocrine-based treatments, BSE-mediated inhibition of MDA-MB-231 tumors indicates broader therapeutic relevance and implies that BSE acts independently of hormone receptor status. This capacity to modulate tumor progression across distinct molecular subtypes emphasizes the potential of BSE as a versatile anticancer agent, particularly in the context of heterogeneous breast cancer.

The clinical relevance of these findings is partly supported by a recent clinical study in patients with breast cancer, in which oral administration of an isothiocyanate-rich broccoli sprout extract resulted in measurable sulforaphane metabolites in breast tissue [35]. Together with the consistent anti-tumor activity observed in both cellular and xenograft models, our findings suggest that BSE and its bioactive component SFN suppress breast cancer progression through coordinated regulation of oxidative stress, mitochondrial dysfunction, cell cycle arrest, and apoptotic signaling pathways. Nevertheless, the clinical application of BSE requires further investigation because sulforaphane bioavailability can vary considerably among individuals and its immunomodulatory activity may have context-dependent effects during cancer progression. Additional studies will be needed to define optimal dosing strategies and to determine whether BSE can improve the efficacy of current breast cancer treatments.

## 5. Conclusions

Overall, our findings indicate that BSE and its bioactive component SFN exert anti-cancer effects through coordinated modulation of oxidative stress, mitochondrial dysfunction, cell cycle arrest, and apoptotic signaling pathways. Owing to their natural origin, sustained systemic exposure, and favorable safety profile observed in this study, BSE-based interventions may represent a promising phytochemical-based approach for breast cancer management. Future studies evaluating their effects on cancer stem cell populations, as well as their potential synergy with existing therapeutic agents, will be important to further elucidate their clinical relevance.

This study has several limitations. First, a standard chemotherapeutic positive control group was not included in the *in vivo* xenograft experiments. Inclusion of such a comparator would allow direct benchmarking of the antitumor efficacy of BSE against clinically used agents. However, given the distinct mechanisms of action, pharmacokinetics, and toxicity profiles between conventional cytotoxic drugs and phytochemical-based interventions, the present study focused on evaluating the intrinsic efficacy and mechanistic basis of BSE. Second, the *in vivo* antitumor effect of pure SFN was not directly evaluated. Although dose selection was guided by comparable systemic exposure between orally administered BSE and pure SFN, the biological activity of SFN *in vivo* may differ due to differences in tissue distribution, chemical stability, and matrix effects. Therefore, further comparative studies are needed to clarify the relative contributions of BSE and SFN to the observed therapeutic effects.

## Data Availability

The data that support the findings of this study are available from the corresponding author upon reasonable request.

## Supplementary Materials

The following supporting information can be downloaded at: https://www.mdpi.com/article/10.3390/antiox15050569/s1, Figure S1: Serum biochemical parameters related to hepatic and renal function after 5 weeks of treatment in MCF7 tumor-bearing mice.; Figure S2: Histopathological assessment of major organs after repeated oral administration of broccoli sprout extract (BSE) in MCF7 tumor-bearing mice.; Figure S3: Individual tumor volumes for mice in each group showing the antitumor effect of oral BSE in breast cancer xenograft models.; Table S1: Pharmacokinetic parameters of sulforaphane (SFN) in rats following intravenous (IV) administration of SFN and oral administration of SFN or broccoli sprout extract (BSE) at different doses.

## Abbreviations

The following abbreviations are used in this manuscript:

BSE	broccoli sprout extract
SFN	sulforaphane
TNBC	triple-negative breast cancer
HER2	human epidermal growth factor receptor 2
ROS	reactive oxygen species
MMP	mitochondrial membrane potential
CHOP	C/EBP homologous protein
CDC2	cell division cycle 2
GRP78	glucose-regulated protein 78
BAD	BCL2-associated agonist of cell death
Mcl-1	myeloid cell leukemia 1
BID	BH3-interacting domain death agonist
Bax	BCL2-associated X protein
Bcl-xL	B-cell lymphoma-extra large
Bcl-2	B-cell lymphoma 2
cyt c	cytochrome c
COX4	cytochrome c oxidase subunit 4
Apaf-1	apoptosis-activating factor 1
PARP	poly(ADP-ribose) polymerase
IV	intravenous
ECL	enhanced chemiluminescence
PMSF	phenylmethylsulfonyl fluoride
ER	endoplasmic reticulum
RT	room temperature
PI	propidium iodide
RIPA	radioimmunoprecipitation assay
PBS	phosphate-buffered saline
MTT	3-(4,5-dimethylthiazol-2-yl)-2,5-diphenyltetrazolium bromide
DAPI	4′,6-diamidino-2-phenylindole
TUNEL	terminal deoxynucleotidyl transferase dUTP nick end labeling
NAC	N-acetylcysteine
