# Supplementary material for: Anticancer Effects of Broccoli Sprout Extract and Sulforaphane Through ROS-Induced MAPK Pathway in Breast Cancer Cells and Xenografts
Source: Antioxidants (Basel). 2026 Apr 30;15(5):569. doi: 10.3390/antiox15050569 (PMC13203652; doi:10.3390/antiox15050569)
Supplement: Supplementary file 1 [file antioxidants-15-00569-s001.zip › antioxidants-4240630-supplementary.pdf]

Supporting Information

Anticancer effects of broccoli sprout extract and sulforaphane through ROS-induced MAPK pathway in breast cancer cells and xenografts

Seung-On Lee [1,†], Ji Eun Yu [1,2,†], Laxman Subedi [1], Susmita Phuyal [1], Arjun Dhwoj Bamjan [1], Goo Yoon [2], Sang Hoon Joo [3], Suk-Jung Oh [4], Jin Woo Park [1,2,*], Jung-Hyun Shim [1,2,5,*]

[1]Department of Biomedicine, Health & Life Convergence Sciences, BK21 Four, College of Pharmacy, Mokpo National University, Muan 58554, Republic of Korea

[2]Department of Pharmacy, College of Pharmacy, Mokpo National University, Muan 58554, Republic of Korea

[3]College of Pharmacy, Daegu Catholic University, Gyeongsan 38430, Republic of Korea

[4]Research & Development, Ecoworld Pharm Co. Ltd., Damyang 57304, Republic of Korea

[5]The China-US (Henan) Hormel Cancer Institute, Zhengzhou, Henan, 450008, P.R. China

† These authors contributed equally to this work.

* Correspondence: Tel: +82-61-450-2704, e-mail: jwpark@mokpo.ac.kr (JW. P.); Tel: +82-61-450-2684, e-mail: s1004jh@gmail.com (J-H. S.)

Running title: Sulforaphane ROS-MAPK effects in breast cancer

Supplementary Table

Table S1. Pharmacokinetic parameters of sulforaphane (SFN) in rats following intravenous (IV) administration of SFN and oral administration of SFN or broccoli sprout extract (BSE) at different doses.

**Test material**	**SFN-IV**	**SFN-Oral**	**BSE (5)**	**BSE (10)**	**BSE (20)**
Administration route	IV	Oral	Oral	Oral	Oral
SFN dose (mg/kg)	1	5	5	10	20
T_{max} (h)		2.00 ± 0.00	2.00 ± 0.00	2.00 ± 0.00	2.00 ± 0.00
$T_{1/2}$ (h)	1.48 ± 0.93	4.27 ± 0.30	4.74 ± 0.93	9.11 ± 5.65	10.5 ± 3.02
C_{max} (ng/mL)	100 ± 16.7	89.6 ± 7.66	88.5 ± 7.92	154 ± 3.39	324 ± 15.0
AUC_{last} (ng·h/mL)	99.1 ± 7.38	406 ± 31.1	445 ± 5.16	848 ± 13.0	1664 ± 27.6
AUC_{inf} (ng·h/mL)	99.7 ± 7.80	425 ± 36.2	4.62 ± 5.52	1001 ± 94.0	1928 ± 128
Bioavailability (%)	100	82.0 ± 6.28	89.9 ± 1.04	85.6 ± 1.32	83.7 ± 1.39

T_{max}, time to reach maximum plasma concentration; $T_{1/2}$, half-life of plasma concentration; C_{max}, maximum plasma concentration; AUC_{last}, area under the plasma concentration-time curve from zero to the time of the last measurable plasma concentration; AUC_{inf}, area under the plasma concentration-time curve from zero to infinity. Each value represents the mean ± SD (*n* = 3). Bioavailability (%) = $(AUC_{last,\ oral}/Dose_{SFN,\ oral})/(AUC_{last,\ IV}/Dose_{SFN,\ IV}) \times 100$.

Supplementary Figures 1, 2, and 3

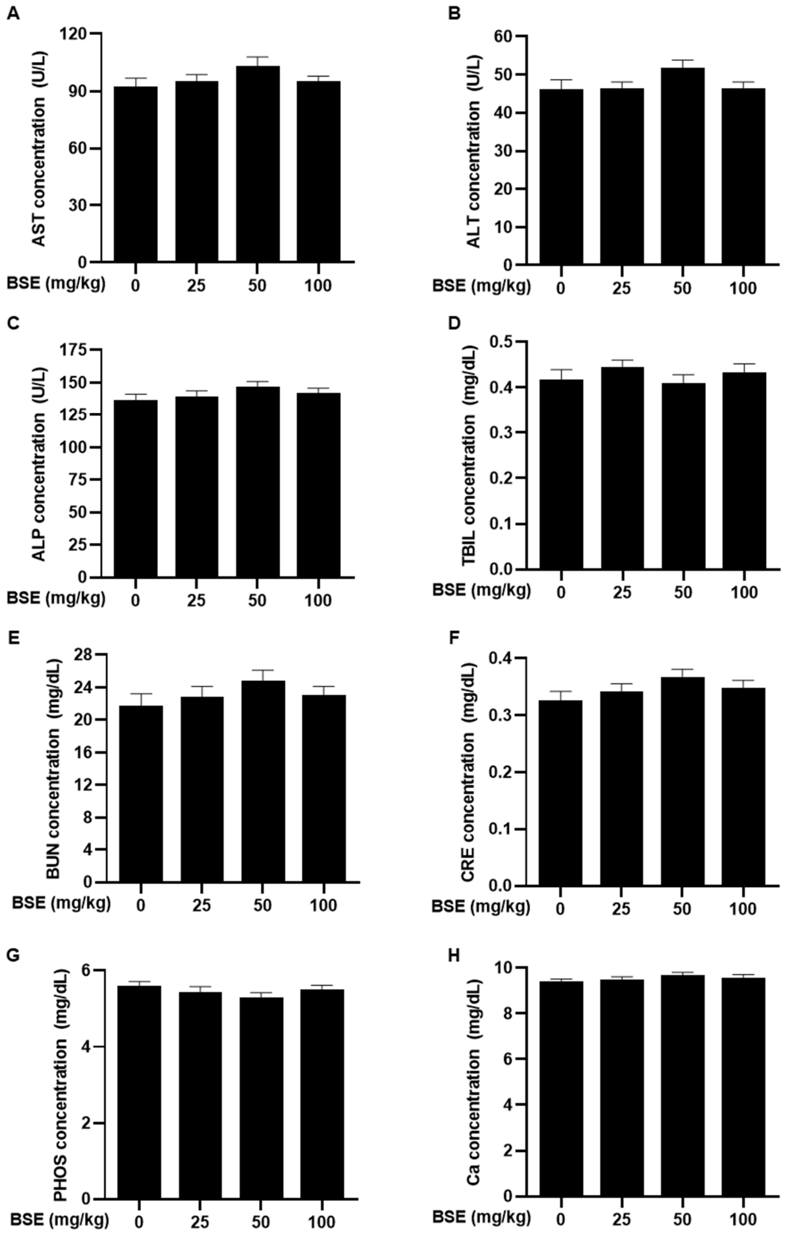

Figure S1. Serum biochemical parameters related to hepatic and renal function after 5 weeks of treatment in MCF7 tumor-bearing mice. (A) Aspartate aminotransferase (AST), (B) alanine aminotransferase (ALT), (C) alkaline phosphatase (ALP), (D) total bilirubin (TBIL), (E) blood urea nitrogen (BUN), (F) creatinine (CRE), (G) inorganic phosphorus (PHOS), and (H) calcium (Ca). Data are presented as mean ± SD (n = 10).

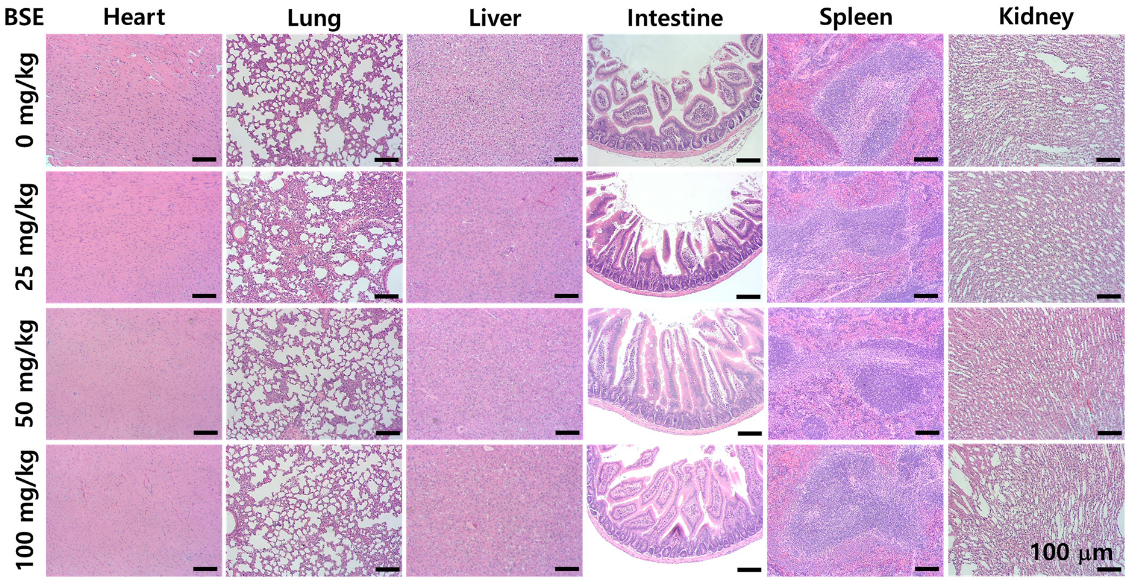

Figure S2. Histopathological assessment of major organs after repeated oral administration of broccoli sprout extract (BSE) in MCF7 tumor-bearing mice. Representative hematoxylin and eosin (H&E)-stained sections of the heart, lungs, liver, intestine, spleen, and kidneys from the control (vehicle) and BSE-treated (oral) groups after 3 weeks of repeated administration. Scale bar: 100 μm.

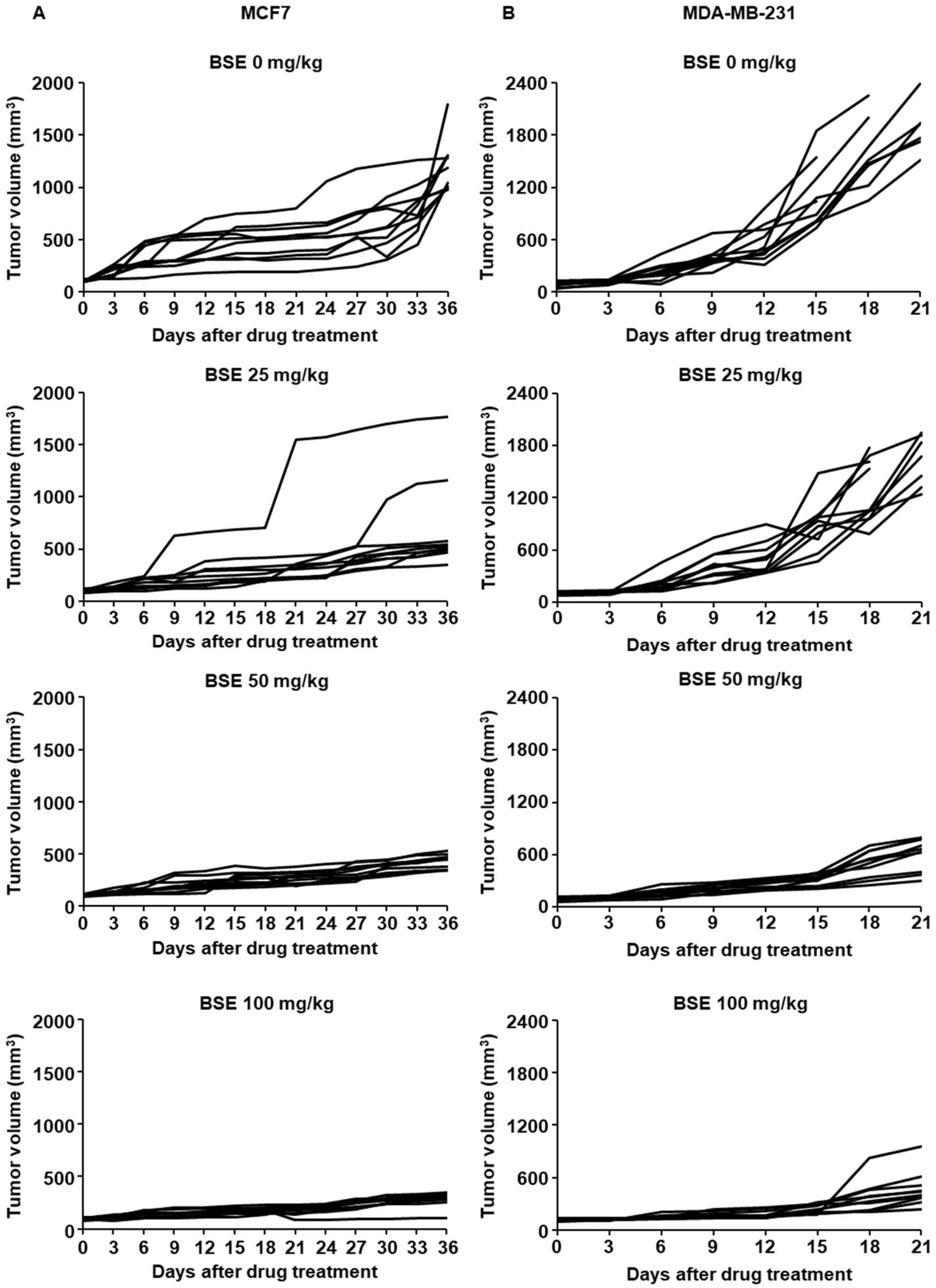

Figure S3. Individual tumor volumes for mice in each group showing the antitumor effect of oral BSE in breast cancer xenograft models. (A) MCF-7 and (B) MDA-MB-231 tumor-bearing mice treated with BSE at doses of 0 mg/kg (control), 25 mg/kg, 50 mg/kg, and 100 mg/kg (*n* = 10).
